# Smoking and healthcare expenditure reductions associated with the California Tobacco Control Program, 1989 to 2019: A predictive validation

**DOI:** 10.1371/journal.pone.0263579

**Published:** 2023-03-16

**Authors:** James M. Lightwood, Steve Anderson, Stanton A. Glantz

**Affiliations:** 1 Department of Clinical Pharmacy, University of California, San Francisco, San Francisco, California, United States of America; 2 JPMorgan Chase & Co., San Francisco, California, United States of America; 3 Analytical Steve Consulting, San Francisco, California, United States of America; 4 Department of Medicine, Center for Tobacco Control Research and Education, Philip R. Lee Institute for Health Policy Studies, University of California San Francisco, San Francisco, California, United States of America; Medical University of South Carolina, UNITED STATES

## Abstract

**Background:**

Previous research used data through 2008 to estimate a model for the effect of the California Tobacco Control Program (CTCP) that used cumulative real per capita tobacco control expenditure to predict smoking behavior (current adult smoking prevalence and mean cigarette consumption per current smoker). Predicted changes in smoking behavior due to the CTCP were used to predict its effect on health care expenditure. This research updates the model using the most recently available data and estimates CTCP program effect through 2019.

**Methods:**

The data used in the previous research were updated, and the original model specification and a related predictive forecast model were re-estimated. The updated regression estimates were compared to those previously published and used to update estimates of CTCP program effect in 2019 dollars.

**Results:**

There was no evidence of structural change in the previously estimated model. The estimated effect of the CTCP program expenditures on adult current smoking prevalence and mean consumption per adult current smoker has remained stable over time. Over the life of the program, one additional dollar per capita of program expenditure was associated with a reduction of current adult smoking prevalence by about 0.05 percentage point and mean annual consumption per adult current smoker by about 2 packs. Using updated estimates, the program prevented 9.45 (SE 1.04) million person-years of smoking and cumulative consumption of 15.7 (SE 3.04) billion packs of cigarettes from 1989 to 2019. The program produced cumulative savings in real healthcare expenditure of $544 (SE $82) billion using the National Income and Product Accounts (NIPA), and $816 (SE $121) billion using the Center for Medicare and Medicaid Services (CMS) measure of medical costs. During this time, the CTCP expenditure was $3.5 billion.

**Conclusion:**

A simple predictive model of the effectiveness of the CTCP program remained stable and retains its predictive performance out-of-sample. The updated estimates of program effect suggest that CTCP program has retained its effectiveness over its 31-year life and produced a return on investment of 231 to 1 in direct CMS medical expenditure.

## Background

The California Tobacco Control Program (CTCP) was established in 1988 when California voters increased the tobacco tax and allocated 20% of proceeds to create a tobacco control program [1]. The program adopted a comprehensive and integrated approach to tobacco use and prevention aimed at changing social norms that previously tolerated and encouraged tobacco use [2], including a media campaign and community programs to encourage effective local tobacco control measures [3]. Previous research published in 2013, ‘The Effect of the California Tobacco Control Program on Smoking Prevalence, Cigarette Consumption, and Healthcare Costs: 1989–2008’ [4] (ECTCP), presented a time series cointegrating regression model that predicted adult current smoking prevalence (‘prevalence’) and mean cigarette consumption per adult current smoker (‘consumption’) as a function of real per capita tobacco control expenditure (‘control expenditure’). Then predicted prevalence and consumption were used to predict two measures of real per capita healthcare expenditure in California: one using the Centers for Medicare and Medicaid Services measure (‘CMS expenditure’) and one using the National Income and Product Accounts (NIPA) measure (‘NIPA expenditure’). The previous research [4] estimated that, from 1989 through 2008, the CTCP cost $2.4 billion ($2.8in 2019 dollars) and had a healthcare expenditure savings of $134 billion in 2010 dollars ($172 billion in 2019 dollars).

There are two reasons for updating the existing estimates. First, updating the estimated effect of the CTCP is an opportunity to validate the out-of-sample performance of the model developed in the previous research. ECTCP [4] modeled the relationships between tobacco control spending, smoking prevalence, mean cigarette consumption per smoker, and healthcare expenditure. Unless there is a major structural break in the underlying relationships, the ECTCP regression estimates should be stable over a longer sample period. Second, if the model estimated in ECTCP [4] remains stable and retains good predictive performance, it is of inherent interest to evaluate the continued effect of the CTCP since 2008.

Simple predictive models alone cannot provide strong evidence for causal effect, or descriptions of the detailed causal chain from tobacco control policy to the many desired outcomes. However, a predictive model that remains stable, and can reliably predict broad outcome measures out-of-sample, can strengthen evidence for causal effects that have been previously established with other study designs and help translate causal analysis into practical program planning [5, 6].

The sample data for the main analysis in the ECTCP ended in 2008. ECTCP included a small out-of-sample forecast validation for the Center for Medicare and Medicaid Services (CMS) measure of healthcare expenditure from the years 2005 to 2008, because new data were released towards the end of that research. That previous out-of-sample validation for the model was limited to a four-year horizon. Validation of the whole model out of the original estimation sample over a longer time horizon will provide a stronger test of model stability and predictive accuracy. The current research uses the most recent data for smoking prevalence (2018), cigarette consumption (2018), and medical expenditure (2014 for the CMS measure, and 2017 for the National Income and Product Accounts [NIPA] measure). The last year of the forecasts used for estimation of program effect is 2019, 31 years after it was implemented in 1989.

## Methods

The research has four goals: (1) Determine how well the previously published model performs for out-of-sample prediction. (2) Re-estimate the model to determine parameter stability over out-of-sample estimation. (3) Determine whether a minor variation of the model (in this case, one that used only lagged explanatory variables) could be developed that was more predictive and more suitable for forecasting (i.e., the dependent variables are functions only of lagged explanatory variables), and (4), if the out-of-sample validation is successful, update estimates of program impact.

The methods consist of the following steps:

1. `Update the data used in ECTCP [4] to those most recently available and check for consistency with the data used in previous research.
2. Re-estimate the identical models used in ECTCP and a purely predictive version of the model:
  - from 1985 to 2008 for comparison to the published ECTCP [4] estimates
  - from 1985 to the most recent data available to update forecasts.
3. Evaluate the estimates and model predictions from Step 2
4. Choose the model which fits the data best to estimate the impact of the CTCP expenditure from 1989 to 2019, the CTCP program effect, compared to the counterfactual of no CTCP expenditure.

### Estimated models

Two models were estimated with the updated data. The first model is identical to the one published in ECTCP [4] and will be referred to as the ‘published model’. The published model is used to validate the previously published results with data not used in the original publication. The second model, referred to as the ‘forecast model’, is the same as the published model except that it includes only lagged explanatory variables. If the forecast model performed well, it would be preferred for estimating the CTCP impact: lagging all explanatory variables reduces the risk of endogeneity between the California and the control state variables and is easier to use for forecasting healthcare. Because the forecast model performed better on the most important measure, it was used for estimation of program effect.

As in ECTCP [4], the models consist of four equations. The first equation in each version of the model (Eq 1) predicts prevalence; the second predicts consumption (Eq 2); the third predicts the National Product and Income Accounts (NIPA) expenditure (Eq 3). the fourth predicts the Centers for Medical and Medicaid Services (CMS) expenditure (Eq 4). The NIPA measure of healthcare expenditure is used for national product and income accounting; it focuses on medical healthcare goods and services. The CMS measure of healthcare expenditure is industry-based, and classifies most expenditure by healthcare provider organizations as healthcare related. One example of the difference between NIPA and CMS measures is that NIPA has stricter rules to avoid double counting sales of intermediate goods and services. A second difference is that CMS includes some services not strictly related to healthcare, but produced by the healthcare industry. The two measures differ in level, but are highly correlated over time [7].

The forecast model chosen to estimate program effect is described below.

#### Forecast model

Current adult smoking prevalence in California:

$$\left(prev_{c,t-1}-prev_{CA,t}\right)=\alpha_{0}+\alpha_{1}\left(EC_{CA,t-1}-EC_{c,t-1}\right)+\alpha_{2}\left(p_{CAt-1}-p_{c,t-1}\right)+\alpha_{3}\left(y_{CA,t-1}-y_{c,t-1}\right)+\varepsilon_{1,t}$$ (1)

Cigarette Consumption per Smoker:

$$\left(cpsa_{c,t-1}-cpsa_{CA,t}\right)=\beta_{0}+\beta_{1}\left(EC_{CA,t-1}-EC_{c,t-1}\right)+\beta_{2}\left(p_{CA,t-1}-p_{c,t-1}\right)+\beta_{3}\left(y_{CA,t-1}-y_{c,t-1}\right)+\varepsilon_{2,t}$$ (2)

NIPA Healthcare Expenditure:

$$n_{CA,t}=\gamma_{0}+\gamma_{1}n_{c,t-1}+\gamma_{2}\left(prev_{c,t-1}-prev_{CA,t-1}\right)+\gamma_{3}\left(cpsa_{c,t}-cpsa_{CA,t-1}\right)+\gamma_{4}\left(y_{CA,t-1}-y_{c,t-1}\right)+\varepsilon_{3,t}$$ (3)

CMS Healthcare Expenditure:

$$h_{CA,t}=\delta_{0}+\delta_{1}h_{c,t-1}+\delta_{2}\left(prev_{c,t-1}-prev_{CA,t-1}\right)+\delta_{3}\left(cpsa_{c,t}-cpsa_{CA,t-1}\right)+\delta_{4}\left(y_{CA,t-1}-y_{c,t-1}\right)+\varepsilon_{4,t}$$ (4)

Where *prev*_*j*, *t*_: Prevalence of current adult smoking (‘prevalence’) in population *j*, for California and control states in year *t*, in percentage points, *cpsa*_*j*, *t*_: Mean cigarette consumption per current adult smoker (‘consumption’) in population *j*, for California and control states in year *t*, in packs/year per smoker, *EC*_*j*, *t*_: Cumulative real per capita tobacco control funding (‘control expenditure’) in population *j*, for California and control states in year *t*, in dollars, *p*_*j*, *t*_: Real price per pack of cigarettes in population *j*, for California and control states in year *t*, in dollars, *y*_*j*, *t*_: Real per capita personal income in population *j*, for California and control states in year *t*, in thousands of dollars, *n*_*j*, *t*_: Real per capita NIPA healthcare expenditure (‘NIPA expenditure’) in population *j*, for California and control states in year *t*, in thousands of dollars, *h*_*j*, *t*_: Real per capita CMS healthcare expenditure (‘CMS expenditure’) in population *j*, for California and control states in year *t*, in thousands of dollars, *F065*_*k*,*t*_: Stationary error terms for equation *k* = 1 to 4, in year *t*, *j*: Index for population *j* = *CA* for California (intervention), *j* = *c* for control state population, *t*: Time index, *t* = 1985 to *T*, and *T* is the most recent observation available.

The dependent variables (*prev*_*c*,*t−1*_ − *prev*_*CA*,*t*_) (Eq 1) and (*cpsa*_*c*,*t−1*_ − *cpsa*_*CA*,*t*_) (Eq 2) are formally equivalent to using California prevalence, *prev*_*CA*,*t*_, and consumption, *cpsa*_*CA*,*t*_, as dependent variables and the corresponding lagged control population variables as explanatory variables with regression coefficients constrained to be equal to one.

Note that we used the term ‘tobacco education’ in previous research rather than ‘tobacco control’ which we use here. Changes in CTCP program effort since the previous research make the word ‘control’ a more accurate representation of total program effort over the whole sample period.

See S1a-S4a Eqs. in the S1 Text for the previously published ECTCP model that was used to evaluate out-of-sample predictions for model stability.

### Data

Data series used for the new estimates were obtained from the original data sources used in ECTCP for 1989 to 2008 whenever possible and updated to the most recent year of data available. There were some minor differences between the updated data set (the one used in ECTCP) due to differences in data availability and methods used for the source data (See S1 Text); however, they made little difference in the estimates. Two differences in the two data sets should be noted. The first is adjustment of the measure of consumption to match per capita consumption to adult smoking prevalence when calculating mean consumption per adult current smoker. This change made little difference in the analysis other than increasing the coefficient of mean consumption in Eq 2 by approximately (1/0.75) = 1.33. The second difference is due to a change in Behavioral Risk Factor Surveillance Survey (BRFSS) methodology in 2011, which potentially affected the level and slope of measured prevalence and consumption. Potential effects of this change were modeled using statistical adjustment to prevent bias in the regression coefficients due to measurement error. See the discussion below, and the S1 Text for details of methods used for the updated data.

The intervention population was the aggregate population of California that was exposed to the CTCP. The control population was the aggregate population of 38 control states that had not had a continuous tobacco control program from 1989 to 2000, [8]. The control states are Alabama, Arkansas, Colorado, Connecticut, Delaware, Georgia, Idaho, Illinois, Indiana, Iowa, Kansas, Kentucky, Louisiana, Maine, Minnesota, Mississippi, Missouri, Montana, Nebraska, Nevada, New Hampshire, New Mexico, North Carolina, North Dakota, Ohio, Oklahoma, Pennsylvania, Rhode Island, South Carolina, South Dakota, Tennessee, Texas, Utah, Vermont, Virginia, West Virginia, Wisconsin, and Wyoming. Estimates of smoking prevalence are not available for all of the 38 control states starting in 1989; data from 13 states were available as of 1984 and all were available by 1994. As a result, each of the 38 control states contributed to the control population as annual estimates of state smoking prevalence became available. The entry of each state into the BRFSS system is considered to be exogenous to its smoking behavior and tobacco control policy. A sensitivity analysis, described below, investigated the possibility of bias due to the choice of control states.

The regression data and coefficient estimates were kept in 2010 dollars to facilitate comparison of regression results to those in ECTCP. The estimated impacts of the CTCP on the value of cigarette sales and healthcare expenditure were converted to 2019 dollars.

The time spans for estimation were determined by data availability. The first year of the times series sample was 1985 for all equations, including one annual lagged observation. Data for tobacco control expenditure were available to 2016 (32 observations) prevalence and consumption to 2018 (34 observations); CMS and NIPA measures of healthcare expenditure to 2014 (30 observations) and 2017 (33 observations), respectively.

### Statistical analysis and estimate of program effect

The estimation methods and the estimation of program effect were the same as in ECTCP [4]. Optimal irrelevant instrumental variables [9] were used to estimate each regression (a first order autoregression) separately with two-stage least squares [4].

Several variables are affected by a break in BRFSS survey methodology in 2011, which produced state-specific changes in measured trends in prevalence. This level change also affected the consumption series, which is a function of prevalence. The break was modeled using the same techniques used for previously published national panel data estimates [10, 11] of the effect of state level smoking behavior on healthcare expenditure. Following the previous research [12, 13], a measurement adjustment model was added to the regression to model the effect of the break in survey methodology. A time series break indicator was added to the explanatory variables to the regression equations for the observations after 2011. The indicator variable was equal to zero before 2011 and one in 2011 and afterward, in order to model the level shift. Two interaction terms were also added—prevalence and consumption times the indicator variable—to model possible changes in trends. Therefore, all estimated regression slope coefficients, forecasts, and program effects are adjusted for the change in BRFSS methodology. See the S1 Text for the regression specification with the measurement adjustment model.

Standard descriptive statistics were used to validate consistency of the updated data with those used in previous research and forecast performance. Diagnostic tests were used to evaluate the updated regression estimates. See the S1 Text for details.

Stata version 16 was used for statistical analysis [14].

### Data validation and model selection

#### Data and model validation to 2008

The updated data were compared to those used in ECTCP from 1989 to 2008. The published model (Eqs. 1 to 4) was re-estimated using the updated data set to 2008 and compared to the published estimates.

#### Estimation of published and forecast models using updated data

The published model (Eqs. 1–4) was re-estimated using the updated data set from 1985 to the latest year of data available. If the regression estimates failed any of the diagnostic tests, the model or estimation techniques were modified to address any discovered problems, or the equation was re-estimated using methods robust to the violation in order to check the reliability of the results.

#### Evaluation of model predictions

Predictions for the following variables were used to evaluate the models:

1. prevalence for California (the dependent variable for Eq 1)
2. consumption for California (the dependent variable for Eq 2)
3. NIPA expenditure for California (the dependent variable for Eq 3)
4. CMS expenditure for California (the dependent variable for Eq 4)

The predictions were compared using the following regression estimates:

1. the regression estimates to 2008 in ECTCP [4]
2. the published model (S1a-S4a Eqs. in S1 Text) estimated using the updated data
3. the forecast model (Eqs. 1 to 4) estimated using the updated data

The original and updated time series were compared using correlation coefficients. The Root Mean Square Error (RMSE) of model predictions of each equation were used as the primary criterion to evaluate model and estimate regression in-sample prediction adequacy. The correlation coefficient was used as the secondary criterion. The relative RMSE and the one-step and multi-step Root Mean Square Model Error (RMSME) were also calculated. The RMSME was calculated for the healthcare equation predictions using the predicted time series for prevalence and consumption. The RMSME was the more important criterion for estimation of CTCP program effect because health expenditure estimates that use predicted prevalence and consumption are needed to evaluate the observed time series versus the counterfactual of no CTCP program.

#### Estimation of program effect

The better predictive model (published model versus forecast model) was used to estimate program effect using the same methods as in ECTCP, which was calculated to 2019 using the last year of available data for the predictor variables (2017). The last year of data for state level tobacco control expenditure is 2016, so the average of the last five years of available data was used for the 2017 observation. Briefly, the program effect was simulated using conditional forecasts that assumed all explanatory variables, other than CTCP expenditure, remained the same in the historical scenario and set tobacco control expenditure to zero in the no expenditure scenario. The simulation estimates of program effect used 50,000 trials and were calculated using the Yasai [15] excel add-in.

### Sensitivity analysis

Three sensitivity analyses were conducted to check the robustness of the estimates.

1. Effect of including additional explanatory variables in all equations.
  We explored the potential for bias in the regression estimates due to omitted explanatory variables between California and control states, such as differences in demographics. A measure of healthcare system capacity, total hospital beds per capita, was also added to the NIPA expenditure (Eq 3) and CMS expenditure (Eq 4) regressions.
2. Choice of the control population.
  The study design of this research requires that the control population not be exposed on average to a tobacco control program of similar design and intensity of the CTCP. This assumption cannot be guaranteed in any predictive model that is useful for forecasting out of the estimation sample into the future. This sensitivity analysis re-estimated Eqs. 1 and 2 using control populations with different levels of exposure to tobacco program effort in order to determine the sensitivity of the results to this assumption for the 38 control states used for the analysis.
3. The effect of a positive discount rate for the effect of CTCP expenditure.
  The ECTCP [4] and other previous research [16] assumed that the effect of one real (inflation adjusted) dollar in CTCP program effort spent today does not decay over time in the future. That is, one dollar spent now will have the same effect five years in the future. This assumption needs to be examined as the sample period increases. Therefore, we conducted a sensitivity analysis of the results for annual discount rates of program effect of 0% to 10%.

See the S1 Text for more detailed descriptions of the sensitivity analyses.

## Results

### Data and model validation to 2008

The correlations between the data used in ECTCP and the updated data set from 1989 to 2009 exceeded 0.97 for all variables in Eqs. 1 to 4 except for personal income, (*y*_*c*,*t*−1_ − *y*_*CA*,*t*−1_), for which the correlation was 0.87. The lower correlation for personal income was due to changes in the Bureau of Labor Statistics (BLS) price index methodology (see S1 Text) [17]. After using the California Department of Finance price indices for California [18], which partially corrected for changes in BLS methods, the correlations between the old and updated personal income variables (*y*_*c*,*t*−1_ − *y*_*CA*,*t*−1_) exceeded 0.97. The correlation was 0.97 for the CMS measure of the healthcare expenditure for California and control states, and 0.99 for the other variables included in the regressions. The lower correlation for the CMS healthcare expenditure was because successive updates of that series do not preserve the exact data values from previous versions. CMS does not appear to document this aspect of their revisions, but is apparent upon visual comparison of downloads of successive revisions.

The diagnostic tests showed no problems with the regressions using the updated data, except for moderate autocorrelation in the residuals for CMS and NIPA expenditure (Eqs. 3 and 4); however, the results showed no statistically or substantively significant change with regression estimators robust to moderate autocorrelation. The recursive estimates, which show how the coefficients evolve over time, were very stable for all the estimated coefficients of both the published and forecast models (See S1 Fig in S1 Text). The stability of the estimated recursive coefficients is important evidence for model stability over time.

The estimated regression coefficients (Table 1) of the ECTCP and updated data for the effect of tobacco control expenditure on prevalence (Eq 1) are almost identical: 0.0497 (SE 0.00347) and 0.0489 (SE 0.0110), with one dollar of the cumulative CTCP per capita expenditure estimated to reduce prevalence by about 0.5% in both the ECTCP and updated estimates (i.e., if smoking prevalence were 20 percent, a one dollar increase in cumulative CTCP per capita expenditure would reduce prevalence from 20% to 19.9%). The estimated effects of one dollar of cumulative expenditure CTCP per capita in 2010 dollars on consumption per smoker are different (1.39 [SE 0.132] vs. 2.11 [SE 0.271]) because the estimate in the new model is normalized by the entire population and the ECTCP estimate was normalized by the adult population (see S1 Text). Adjusting for the change in the denominator of mean consumption per smoker from total population to adults, the coefficient 1.39 from the published estimate is equivalent to 1.39/0.74 = 1.88, which is inside the 95 percent confidence interval of the updated estimate, so the two estimates are consistent.

**Table 1:** Estimated California smoking prevalence, cigarettes per capita, and per capita healthcare expenditure.

Equation	Dependent Variable	Statistic	ECTPC, 2013	ECTCP, published, updated data to 2008	ECTCP, published, updated data whole sample	ECTCP, forecast, updated data, whole sample	Dimension
Model Equations							
1	(*prev*_*c*, *t*_*−prev*_*CA*, *t*_)	*α*_*1*_ *(EC)*	0.0497 (0.00347)	0.0489 (0.0110)	0.0494 (0.00983)	0.0503 (0.0107)	/$ per capita
	(*prev*_*c*, *t*_*−prev*_*CA*, *t-1*_)**						
		*R*^*2*^ (%)	77	76	79	73	
		*r*_*1*_	0.154	0.121	0.156	-0.0230	
2	(*cps*_*c*, *t*_*−cps*_*CA*, *t*_)	*β*_*1*_ *ECF029*	1.39 (0.132)	2.11 (0.271)	2.02 (0.267)	2.23 (0.303)	/$ per capita
	(*cps*_*c*, *t*_*−cps*_*CA*, *t-1*_)**						
		*R*^*2*^ (%)	81	82	79	78	
		*r*_*1*_	0.148	0.132	0.190	-0.0886	
3	*n*_*CA*, *t*_	*γ*_*2*_ *(prev)*	-35.4 (9.85)	-64.3 (16.1)	-69.5 (13.8)	-54.5 (16.7)	$/%point
		*γ*_*3*_ *(cpsa)*	-3.14 (0.786)	-3.16 (0.398)	-3.22 (0.335)	-3.40 (0.433)	$/pack per smoker
		*R*^*2*^ (%)	80	90	92	86	
		*r*_*1*_	0.262	0.389	0.415*	-0.00420	
4	*h*_*CA*, *t*_	*δ*_*1*_ *(prev)*	-67.8 (7.31)	-149 (32.0)	-130 (25.3)	-86.3 (21.5)	$/%point
		*δ*_*2*_ *(cpsa)*	-5.48 (0.928)	-5.59 (0.865)	-5.24 (0.641)	-5.00 (0.573)	$/pack per smoker
		*R*^*2*^ (%)	89	87	93	94	
		*r*_*1*_	0.486*	0.506*	0.499*	0.461*	

*significant at the 5% level.

** specification of dependent variable for the forecast model.

*r*_*1*_: first order autocorrelation coefficient.

*prev*_*j*, *t*_: Prevalence of current smoking in population j, for California and control states in year t,(percentage points).

*cps*_*j*, *t*_: Cigarettes consumption per current smoker in population j, for California and control states in year t, (packs/year per smoker).

*EC*_*j*, *t*_: Cumulative per capita funding in population j, for California and control states in year t, (dollars).

*n*_*j*, *t*_: Per capita healthcare expenditure in population j, for California and control states in year t, (thousands of dollars).

*h*_*j*, *t*_: Per capita healthcare expenditure in population j, for California and control states in year t, (thousands of dollars).

Note: dollar amounts are in 2010 dollars.

The main difference between the ECTCP and updated estimates is a significantly different coefficient for the effect of prevalence on CMS healthcare expenditure in ECTCP [4], -67.8 (SE 7.31), compared to the updated data set, -149 (SE 32.0) (P-value for difference = 0.0134). The coefficients for the effect of consumption, -5.48 (SE .928) compared to -5.59 (SE 0.865) (P-value for difference = 0.931) are similar. The discrepancy in the effect of prevalence on CMS expenditure may be due to the differences in the entire CMS time series following successive updates and differences in the updated time series for real per capita personal income. The regression coefficients for prevalence and consumption were not different at the five percent significance level for the NIPA expenditure.

### Estimation of ECTCP model using updated data and forecast model

When estimated over the whole sample, there were some potentially influential observations. However, robust regression estimates that reduced the influence of large residuals produced only statistically insignificantly different coefficient estimates of no substantive significance. Therefore, influential observations do not appear to be a problem.

### Evaluation of model predictions

The original published ECTCP estimates and updated estimates performed well in predicting the extended data set: The multi-step out-of-sample RMSEs of the ECTCP model estimates ranged from 6 percent (prevalence) to 100 percent (CMS expenditure) higher than those RMSEs of the updated models. This performance is considered good for three reasons. First, the RMSEs of the ECTCP estimates include out-of-sample predictions while those of the new estimates are in-sample. Out-of-sample predictions will usually be higher than in-sample predictions in relatively small samples. Second, most of the increase in RMSEs for the ECTCP estimates occur at the time of the structural break in the BRFSS data, which cannot be handled by the ECTCP model that was estimated before the break. Third, there is no steadily increasing trend in RMSE for the ECTCP estimates over time, which would be expected if there were a structural break in the model. If there were a breakdown in the cointegrating relationship after 2008, then RMSEs would be expected to increase exponentially over time. The correlations between predictions and observations were close to the models estimated with the updated data (S1 File, S2 and S3 Tables in S1 Text).

The RMSEs for the ECTCP model (S1 File, S2 Table in S1 Text), re-estimated with updated data are slightly smaller for prevalence and consumption (0.737 and 21.23, respectively) than those for the re-estimated forecast model (0.852 and 23.7). Neither the published nor the forecast model dominated, in terms of the RMSE, for healthcare expenditure equations that used observed smoking prevalence and cigarette consumption per smoker. The RMSE for the forecast model was smaller than the published ETCP model for both CMS and NIPA healthcare expenditure equations that used predicted smoking prevalence and cigarette consumption per smoker. For NIPA healthcare expenditure, the RMSMEs for the forecast model and published model were 79.3 and 84.6, respectively. For CMS healthcare expenditure, the RMSMEs were 130 and 123, respectively (S2 Table in S1 Text). The RMSMEs for the healthcare equation models that used predicted prevalence and consumption are more relevant for program evaluation because estimation of program effect depends on predicting health expenditure under two different scenarios for predicted prevalence and consumption over time. The correlations between observed and predicted dependent variables (S3 Table in S1 Text) are higher for the forecast model than the published ECTCP model, except for the prevalence and CMS expenditure with predicted prevalence and consumption, where the ECTCP model was slightly higher. Therefore, the forecast model was chosen for the final model used to update the estimates of the impact of the CTCP program. For all regression estimates, the relative RMSE of predictions of prevalence was less than 6 percent, for consumption less than 13 percent, for NIPA expenditure less than 6 percent, and for CMS expenditure less than 4 percent.

### Updated model estimates

The estimated regression coefficients of the final forecast model that were used to estimate the CTCP effect are shown in Table 1. In the updated estimates of the forecast model, one additional 2010 dollar of cumulative CTCP per capita expenditure reduces prevalence by 0.0503 (SE 0.0107) percentage point and consumption by 2.23 (SE 0.303) packs The estimate in ECTCP reduced consumption by 1.39 (SE 0.132) packs (P-value for difference = 0.01) in ECTCP. Adjustment for the change in the denominator used to calculate consumption from total to adult population results in an estimate for the forecast model of 1.39/0.74*2.22 = 1.88, which is inside the 95 percent confidence interval of the updated forecast model estimate: there is no statistically significant difference.

In 2010 dollars, a reduction in prevalence of one percentage point reduces real per capita NIPA and CMS expenditure by $54.5 (SE $16.7) and $86.3 (SE $21.5), respectively. A reduction in consumption of one pack per year reduces real per capita NIPA and CMS expenditure by $3.40 (SE $0.433) and $5.00 (SE $0.573), respectively. The model fit remains good for predictions for NIPA and CMS expenditure (Eqs. 3 and 4, respectively) when predicted prevalence and consumption are substituted for their observed values, respectively. In the updated estimates for NIPA and CMS expenditure, none of the estimated regression coefficients were statistically or substantively different than the published ECTCP estimates.

S2 and S3 Figs in S1 Text show the observed and predicted prevalence and consumption for California and the difference between California and the control states. S4 Fig in S1 Text shows the NIPA and CMS per capita healthcare expenditure for California. The model estimates and predictions follow the observed data closely.

### Estimation of CTCP program effect

**Fig 1:**
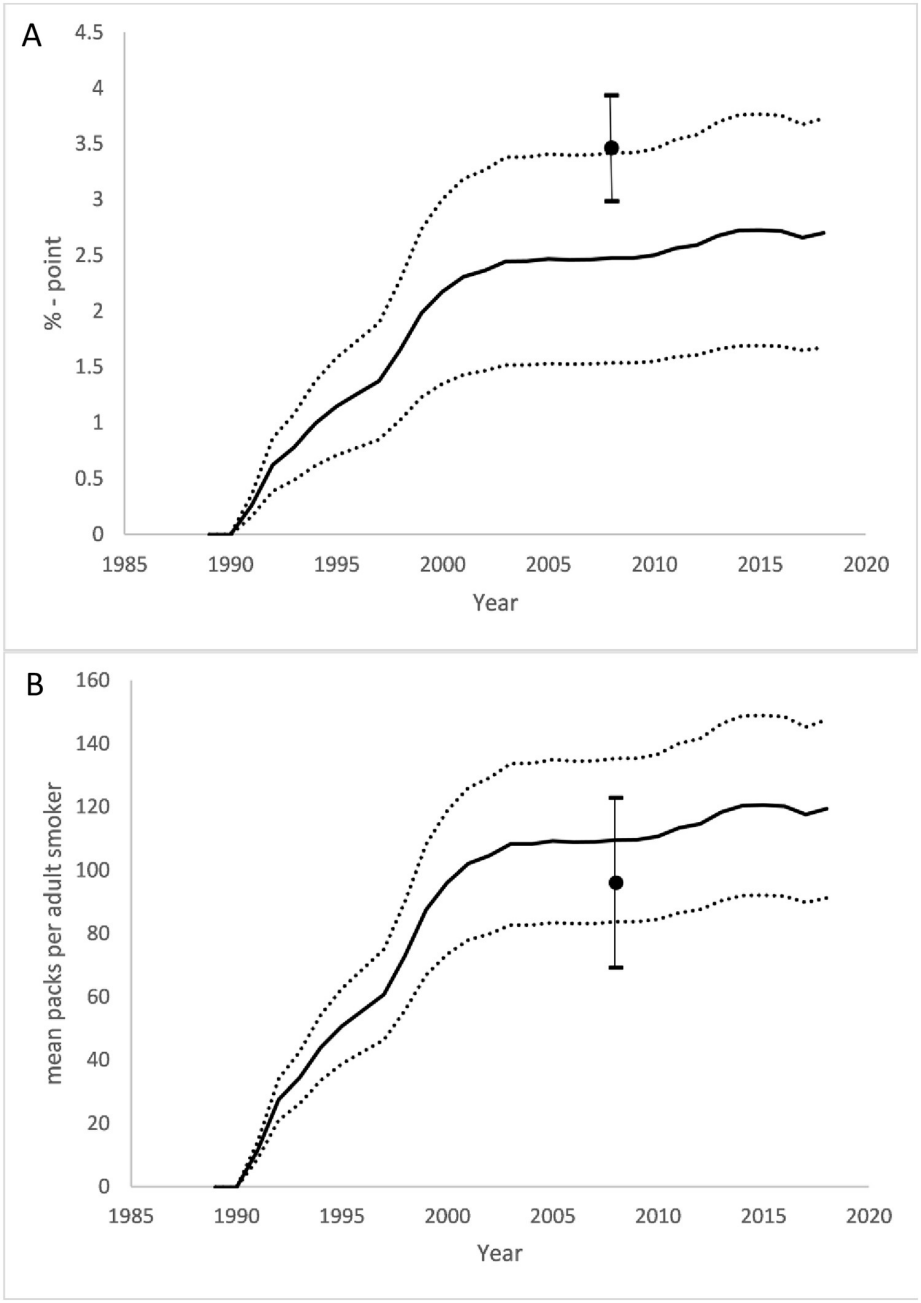
Reductions in smoking attributable to the CTCP. (A) Adult smoking prevalence. (B) Mean packs per current smoker. Black line: mean; Dotted lines: lower and upper 95% confidence intervals for prediction; Black dots and error bars: point and 95% confidence interval reported in the ECTCP [4] 2013 publication.

**Fig 2:**
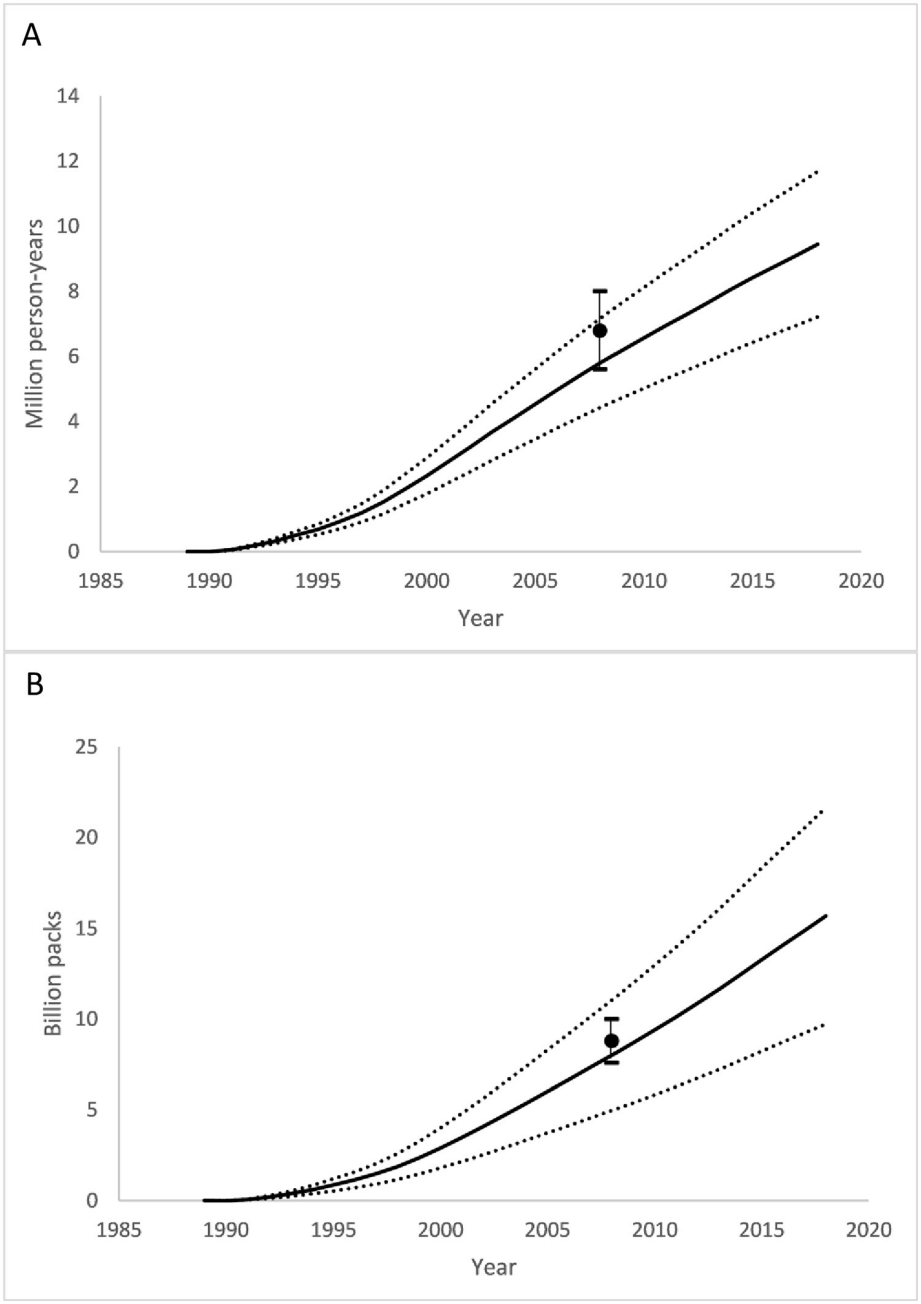
Cumulative reductions in smoking attributable to the CTCP. (A) Person years of smoking. (B) Packs of cigarettes. Black line: mean; Dotted lines: lower and upper 95% confidence intervals for prediction; Black dots and error bars: point and 95% confidence interval reported in ECTCP [4] 2013 publication.

**Fig 3:**
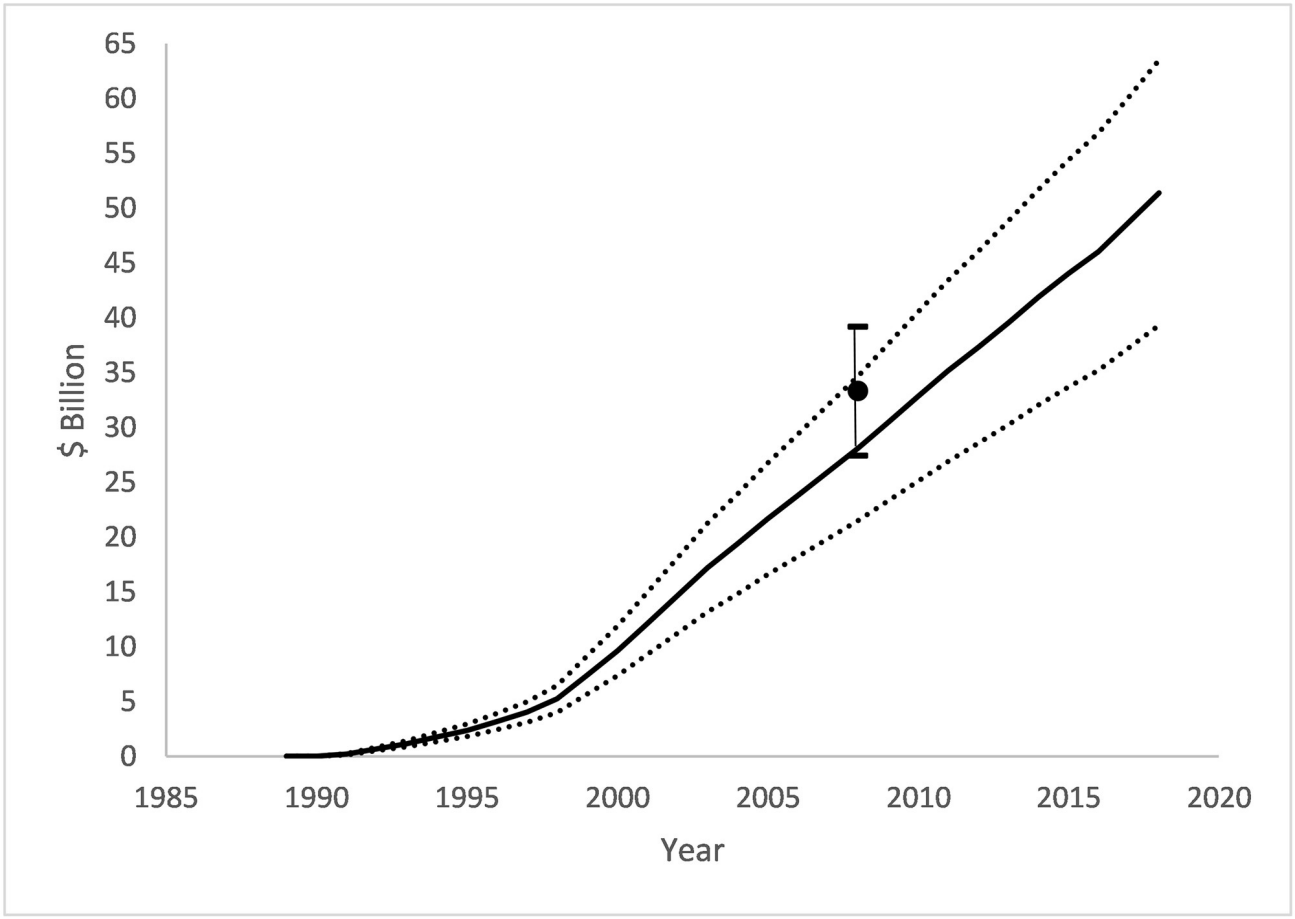
Cumulative reductions in value of pre-tax cigarette sales attributable to the CTCP. Black line: mean; Dotted lines: lower and upper 95% confidence intervals for prediction; Black dot and error bar: point and 95% confidence interval reported in ECTCP [4] 2013 publication. Note: vertical axes are real 2019 dollars.

From 1989 to 2019, the CTCP program reduced annual prevalence by 2.70 (SE 0.524) percentage points and consumption by 119 (SE 14.4) packs/year (Fig 1), compared to the counterfactual of no program. The program was estimated to have prevented 9.45 (SE 1.04) / million person-years of smoking and reduced cumulative consumption by 15.7 (SE 3.04) billion packs of cigarettes (Fig 2). The cumulative value of prevented cigarette sales is $51.4 (SE $6.16) billion (2019 dollars) in pre-tax sales (Fig 3).

**Fig 4:**
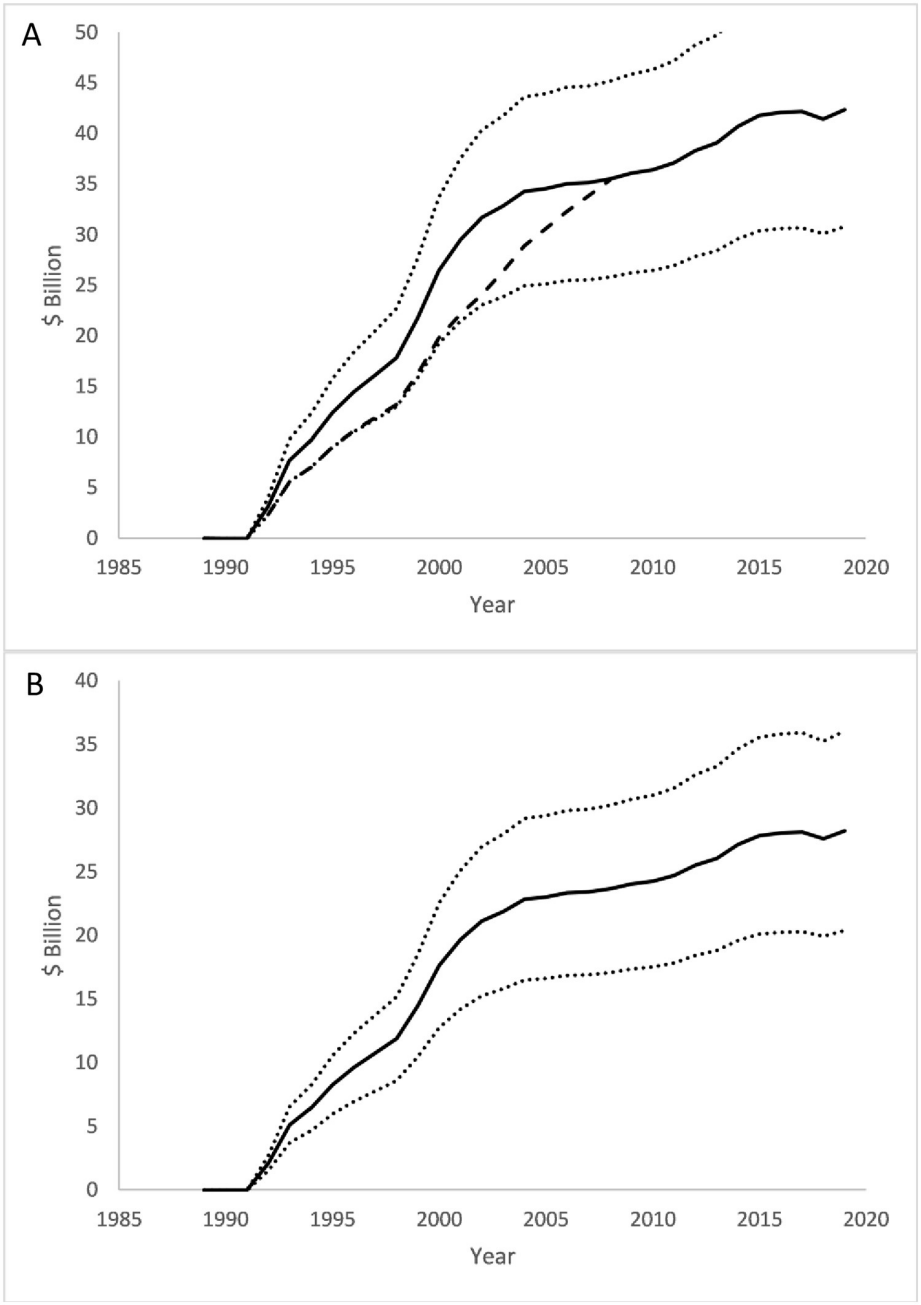
Annual total savings in real per capita healthcare expenditure attributable to the CTCP. (A) CMS measure. (B) NIPA measure. Black line: mean; Dotted lines: lower and upper 95% confidence intervals for prediction. Dashed black line in (A): estimated savings from ECTCP [4]. Note: vertical axes are real 2019 dollars.

**Fig 5:**
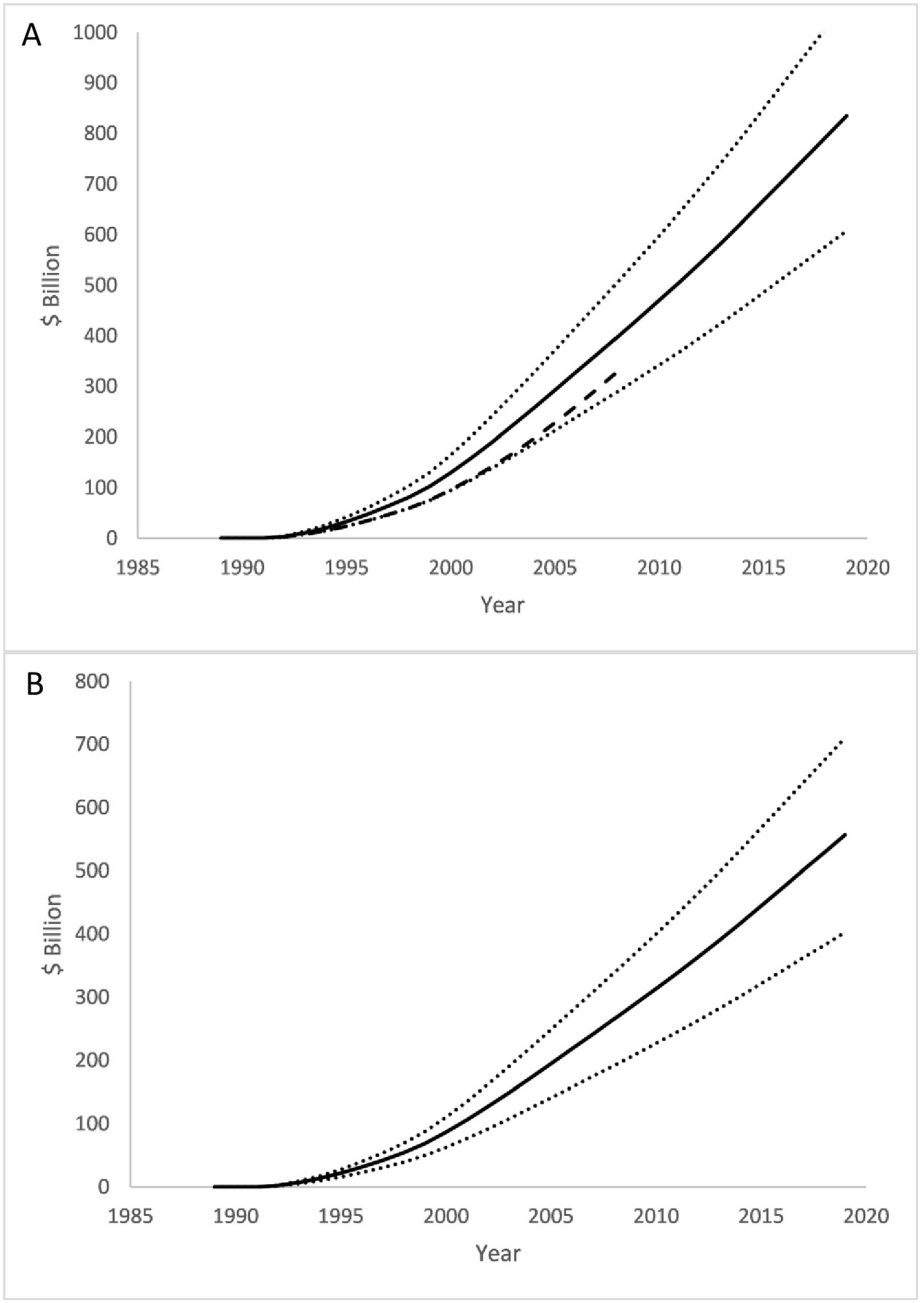
Cumulative savings in real health care expenditure attributable to the CTCP. (A) CMS measure. (B) NIPA measure. Black line: mean; Dotted lines: lower and upper 95% confidence intervals for prediction. Dashed black line in (A): estimated savings from ECTCP [4]. Note: vertical axes are real 2019 dollars.

In 2019, real per capita CMS and NIPA healthcare expenditure was reduced by $42.3 (SE $5.89) billion and $28.2 ($4.00) billion, respectively (Fig 4). From 1989 to 2019, the program was estimated to have produced cumulative savings in real NIPA expenditure of $544 (SE $81.5) billion and in CMS expenditure of $816 (SE $121) billion (Fig 5).

### Sensitivity analysis

1. Effect of including additional explanatory variables in all equations.
  Only two cases produced statistically significant differences in the estimated coefficients of policy interest. The addition of a principal component, which described a dimension of the difference between California compared to the control states that consisted of increased Black, Hispanic population, poverty rate, and decreased Medicaid enrollment, changed the coefficient (*α*_1_) for prevalence (Eq 1) from 0.0503 (SE 0.0107) to 0.0848 (SE 0.0366) (P for difference = 0.668). Addition of the state poverty rate and elderly changed the coefficient (*β*_1_) for CMS expenditure (Eq 4) from -5.00 (SE 0.573) to -3.12 (SE 0.614) (P for difference = 0.025). The practical significance of these results is unclear. Dozens of tests of statistical significance were conducted for inclusion of additional variables and changes in estimated coefficients, and no results would survive standard approaches to adjustment for multiple testing, including methods more powerful than the Bonferroni adjustment (such as the Hochberg adjustments). These results may be due to in-sample overfitting. Stronger evidence is needed for a more definitive conclusion, such as out-of-sample performance of the models with these additional variables.
2. Choice of the control population.
  The estimates of program effect using the original 38 control states are similar to those obtained when using control populations that were exposed to low per capita tobacco control expenditure throughout the sample period.
3. The effect of a positive discount rate for the effect of CTCP expenditure.
  The sensitivity analysis on discount rates was inconclusive when based on summary measures of fit, a result that agrees with other attempts to determine the best discount rate [19]. The sensitivity analysis strongly suggests that a discount rate of zero, or much more likely very close to zero, is appropriate for the effect of tobacco control expenditure on prevalence. For consumption, an annual discount rate of 10 percent or less for tobacco control expenditure may be appropriate for both California and the control population. Increasing the annual discount rate of control expenditure from zero increases the estimates of program effect and total program impact. Using a discount rate of zero produces lower bound estimates.

See S1 Text for detailed results of the sensitivity analyses.

## Discussion

### Evolution of CTCP program impact over time

The regression estimates of the ECTCP model [4] remain stable over the updated data, which extends six to nine years beyond the original sample period of 1989 to 2008, and the original model predictions are satisfactory out-of-sample, and particularly good in terms of correlation with the observed data. The updated data produce estimates of CTCP effect that are substantively and statistically consistent with the ECTCP estimates published in 2013, but over a longer time period. The forecast model regression estimates using the updated data are neither statistically nor practically different from the published ECTCP estimates.

The increases in annual CTCP program impact on prevalence (Fig 1A), mean consumption (Fig 1B), and NIPA and CMS healthcare expenditure (Fig 4A and 4B, respectively) are lower in the updated forecast model estimates after 2005 than those published in ECTC, despite the regression estimates being similar. This change appears to be driven by a reduction in the difference between California and control real annual per capita tobacco control expenditure following 2005, which fell from an average of $4.89 (in 2019 dollars) from 1989 to 2005 to $2.57 in the following years. The effect of this drop in the last 15 percent of the sample would not greatly affect the published estimates in ECTC because they occurred in the last three years of the sample. The vast majority of the control states continued to have very low, though slowly growing, per capita tobacco control expenditure; most of the change in the difference in per capita spending between California and control states is due to reductions in annual real per capita tobacco control expenditure in California. This change in program funding shows that the regression results and estimates of CTCP program effect are robust to changes in the properties of the time series out-of-sample. It also indicates that reductions in the growth in total savings attributable to the program are mostly caused by reduction in program effort not the effect of each real per capita dollar spent. The policy implication of this finding is that cuts to the tobacco control program are rapidly reflected in lower reductions in smoking behavior.

### Related research

Three recent articles were found that are comparable to the approach taken here [8, 19, 20], except that they modeled smoking behavior using the one-dimensional measure of per capita cigarette consumption rather than the two-dimensional measure of smoking prevalence and consumption per smoker. As in this research, these studies model smoking behavior as a function of tobacco control expenditure and taxation or prices, all downstream effects of tobacco control expenditure, except its potential influence on taxes, are omitted as mediating variables. The forecast model regression results for Eqs. 1 and 2 imply that one (real 2010) dollar of control expenditure reduces per capita cigarette consumption by 0.658 (SE 0.0896) packs.

Farrelly et al. [19] find that expenditure of one (real 2010) dollar of control expenditure reduces per capita cigarette sales by 0.476 (SE 0.0984), which is not different from our results at the five percent significance level (P for difference = 0.172). Taurus et al. [20] find that the reduction in per capita consumption is 0.714 (SE 0.288) packs, which is also not different from our results (P for difference = 0.850). Farrelly et al. [19] present results with per capita consumption modeled as a function of cumulative expenditure (i.e., assuming a zero discount rate), current annual expenditure (an annual discount rate equal to one), and lagged annual expenditure. As with our results, the estimated effects of tobacco control expenditure on smoking behavior tend to increase as the discount rate is increased. Farrelly et al. [19] found that using different discount rates had similar explanatory power, which is consistent with our difficulty in determining the most likely discount rates using RMSE alone. However, they present no regression diagnostics, so it is impossible to evaluate the regressions based on violations of the usual assumptions for regression analysis, as we do. See S1 Text for calculations to compare the results of Farrelly et al. [19] and Taurus et al. [20] to these estimates.

Abadie et al. [8] developed a synthetic control matching algorithm to estimate the total CTCP effect on California per capita cigarette consumption from 1989 to 2000, and found that the CTCP produced a reduction in per capita cigarette consumption of 24% to 25%, which was statistically significant at the 5% significance level using a 2-tailed test. Our updated model predicts a 23% reduction (90% prediction interval for the mean reduction: 9.5%, 29%) in per capita cigarette consumption, as calculated by the simulation program used for the estimated program effect. The similarity of the estimated effect may be explained by the similarity of both approaches to finding appropriate control populations. Abadie et al. matches on state per capita GDP (which corresponds to per capita personal income), cigarette price, and per capita cigarette consumption at three different years prior to the beginning of the CTCP (corresponding to regression adjustment for control population trends), as well as per capita beer consumption, and the proportion of young adults in the population. Abadie et al. do not report any statistics on the relative importance of the matching variables, but except for the last two variables, it reflects an approach to comparing intervention and control populations that is similar to that of this research.

Max et al. [21] use a micro-econometric model with individual level cross sectional data and finds that increased prevalence of current and former smoking increased per capita healthcare expenditure are roughly consistent, given the differences in study design and measure of cost between the two studies.

### Limitations

This analysis, like that in ETCTP [4], is a retrospective study that uses historical observational data, and therefore has the limitations of that study design, particularly with regard to establishing causal relationships. This approach cannot supply a detailed structural analysis of the chain of causation from the effect of specific program elements (e.g., media campaigns, smokefree or youth access policies, smoking cessation programs) on the many mediating variables that directly affect the outcome variables. Simple predictive models that are useful for forecasting and program planning cannot provide detailed analysis of the multiple causal chains from implementation of specific program elements to the large number of very specific outcomes (e.g., in different sub-populations) that a public health program should achieve.

This analysis takes advantage of state level characteristics to estimate the total program effect on two very broad outcome measures: prevalence of adult current smoking and mean cigarette consumption in adult current smokers. The simple model used here for California may not be appropriate for the analysis of the effectiveness of other state tobacco control programs. For example, an analysis of the Arizona program and panel data estimates for the 50 states and D.C. required adjustment for state level demographic characteristics [11, 22]. Model specification must be determined on a case-by-case basis.

These estimates combine both long and short run effects of the CTCP, and therefore should be interpreted in the context of the historical development of the CTCP program and its effect on smoking behavior. To give an extreme example for illustration, the model would not produce accurate predictions of the effect of a sudden change in one year from 1989 to 2019 levels of smoking prevalence in California on healthcare expenditure. This caution is partly due to the basic principle that short-run dynamic predictions of a regression should not be extrapolated far outside of the sample data, and these results should interpreted in the context of scenarios that at least roughly approximate the history of the CTCP. A more detailed analysis of the time evolution in cointegrated relationships from program implementation to outcomes would require separate estimates of short and long run effects that are difficult to estimate more precisely in a time series analysis with a relatively small number of annual observations. However, for a model designed primarily for predictive accuracy the combined estimate of the short and long run effects are typically more stable and have better predictive performance [23].

The model also estimates healthcare expenditure for cigarette, not cigar smoking, oral tobacco us, and e-cigarettes. By 2019, however, the tobacco market was changing, with the growing popularity of new tobacco products, particularly e-cigarettes. Unfortunately, there is only limited state-by-state data available on e-cigarette use (The BRFSS only has data for California for 2016 and 2017 [24]). Research using similar methodology finds that the healthcare expenditure attributable to cigar and oral tobacco use are a small fraction of that due to cigarette smoking [25–27], so substantial bias in the estimates is unlikely. As of 2017, adult e-cigarette use was well below cigarette use and nearly half the e-cigarette users were continuing to smoke cigarettes at the same time (Nationally: sole cigarette use prevalence: 14.1%, dual use: 2.3%, sole e-cigarette: 2.5%; California: cigarettes only: 10.8%, dual use: 1.5%, sole e-cigarette: 2.1%.[28] We conducted an exploratory analysis of the potential effects of including trends in e-cigarette use in California and the control states using available data and found that it produces no substantially or statistically significant differences to the estimates (See S1 Text for details).

As of December 20, 2021, there were no population-based estimates of the healthcare expenditure attributable to vaping and e-cigarette use, perhaps due to their short histories. The healthcare expenditure attributable to these products is an important area of future research.

The results of this research should also be interpreted in the context of national tobacco control program efforts. National programs and studies in selected states have either been too small in scale or have affected California and control populations similarly over time, so they do not have a noticeable effect on the estimates of our model, because tobacco control expenditures are mostly are driven by state tax receipts and, and to a smaller extent, by Centers for Disease Control and Prevention aid to state programs. However, these national programs have contributed to reductions in smoking in both California and the control state populations, even if their effects cannot be estimated by the methodology used in this study. There is convincing evidence that multi-state and national programs such as the National Cancer Institute ASSIST study [29], Food and Drug Administration anti-tobacco education programs [30, 31], FDA regulatory actions [32], and the American Legacy Foundation/Truth Initiative programs [33] have also been effective.

## Conclusion

The model and results of the ECTCP analysis in 2013 are stable, and generalize ten years out of the sample used for the previously published estimates [4]. Measures of forecast error are stable over the out-of-sample period. The CTCP continues to have similar effectiveness to that estimated in 2013 per each program dollar spent. The total impact of the program was growing at a slower rate since 2008 mainly because of lower intensity of real per capita funding for the program compared to previous years. Increasing funding would be predicted to increase the impact on smoking and healthcare expenditure savings.

The updated model estimates suggest that between 1989 and 2019, the CTCP has prevented a total of 9.5 million person-years of smoking and 15.7 billion packs of cigarette consumption (worth $51.4 billion in pre-tax sales to the tobacco companies), and saved between $544 billion (NIPA) and $816 billion (CMS) in healthcare expenditure. The total cost of the CTCP over this period was $3.5 billion, resulting on a return on investment of $231 for every $1 of program expenditure.

## Supporting information

S1 Text (DOCX)

S1 Data Data file for final results. (XLSX)

S1 File Stata commands for final results. (DOCX)

## References

[1] Glantz S, Balbach E. Tobacco War: Inside the California Battles. Berkeley: University of California Press; 2000. 336 p.

[2] Tobacco Control Section. A Model for Change: The California Experience in Tobacco Control. Sacramento, CA: California Department of Health Services; 1998.

[3] California Tobacco Control Branch. California Tobacco Control Branch: Toolkits and Manuals Sacramento, CA: California Health and Human Services Agency, California Department of Public Health; 2021 [https://www.cdph.ca.gov/Programs/CCDPHP/DCDIC/CTCB/Pages/ToolKitsAndManuals.aspx.

[4] Lightwood J, Glantz S. The Effect of the California Tobacco Control Program on Smoking Prevalence, Cigarette Consumption, and Healthcare Costs: 1985–2008. PLoS ONE. 2013;8(2):e47145. 23418411 10.1371/journal.pone.0047145 PMC3572143

[5] Buhlmann P. Toward causality and improving external validity. Proc Natl Acad Sci U S A. 2020;117(42):25963–5. doi: 10.1073/pnas.2018002117 33046646 PMC7584988

[6] Shmueli G. To Explain or to Predict? Statistical Science. 2010;25(3):289–310.

[7] Kornfeld R. Health Care Expenditures in the NHEA and GDP. National Economic Accounts Data Users Conference; June 7; Washington DC. Washington DC: Bureau of Economic Analysis; 2011. p. 1–17.

[8] Abadie A, Diamond A, Hainmueller J. Synthetic control methods for comparative case studies: Estimating the effect of California’s Tobacco Control Program. Journal, American Statistical Association. 2010;105(490):493–505.

[9] Phillips PCB. Optimal estimation of cointegrated systems with irrelevant instruments. Journal of Econometrics. 2014;178 part 2:210–24.

[10] Lightwood J, Anderson S, Glantz SA. Predictive validation and forecasts of short-term changes in healthcare expenditure associated with changes in smoking behavior in the United States. PLoS One. 2020;15(1):e0227493. doi: 10.1371/journal.pone.0227493 31945079 PMC6964879

[11] Lightwood J, Glantz SA. Smoking Behavior and Healthcare Expenditure in the United States, 1992–2009: Panel Data Estimates. PLoS Med. 2016;13(5):e1002020. doi: 10.1371/journal.pmed.1002020 27163933 PMC4862673

[12] Centers for Disease C, Prevention. Methodologic changes in the Behavioral Risk Factor Surveillance System in 2011 and potential effects on prevalence estimates. MMWR Morb Mortal Wkly Rep. 2012;61(22):410–3. 22672976

[13] Pierannunzi C, Hu SS, Balluz L. A systematic review of publications assessing reliability and validity of the Behavioral Risk Factor Surveillance System (BRFSS), 2004–2011. BMC Med Res Methodol. 2013;13:49. doi: 10.1186/1471-2288-13-49 23522349 PMC3622569

[14] StataCorp LP. Stata version 16. College Station, Texas2019.

[15] Eckstein J, Riedmuller S. Yasai. New Brunswick, NJ: Rutgers Business School, Rutgers University; 2019.

[16] Lightwood JM, Dinno A, Glantz SA. Effect of the California tobacco control program on personal health care expenditures. PLoS Medicine. 2008;5(8):e178. doi: 10.1371/journal.pmed.0050178 18752344 PMC2522256

[17] Consumer Price Index: History, Handbook of Methods: Bureau of Labor Statistics; 2021 [updated November 24, 2020. https://www.bls.gov/opub/hom/cpi/history.htm.

[18] Demographics: Estimates: Demographics Research Unit, Department of Finance, State of California; 2020 https://www.dof.ca.gov/forecasting/demographics/Estimates/.

[19] Farrelly MC, Pechacek TF, Chaloupka FJ. The impact of tobacco control program expenditures on aggregate cigarette sales: 1981–1998. Journal of Health Economics. 2001;22(203):843–59. 10.1016/S0167-6296(03)00057-2 12946462

[20] Tauras JA, Xu X, Huang J, King B, Lavinghouze SR, Sneegas KS, et al. State tobacco control expenditures and tax paid cigarette sales. PLoS One. 2018;13(4):e0194914. doi: 10.1371/journal.pone.0194914 29652890 PMC5898722

[21] Max W, Sung HY, Shi Y, Stark B. The Cost of Smoking in California. Nicotine Tob Res. 2016;18(5):1222–9. doi: 10.1093/ntr/ntv123 26156629

[22] Lightwood J, Glantz S. Effect of the Arizona tobacco control program on cigarette consumption and healthcare expenditures. Social Science and Medicine. 2011;72(2):166–72. doi: 10.1016/j.socscimed.2010.11.015 21168248 PMC3603372

[23] Hsiao C, Fujiki H. Nonstationary time-series modeling versus structural equation modeling: with an application to Japanese money demand. Monetary and Economic Studies. 1998;May.

[24] Centers for Disease Control and Prevention (CDC). Behavioral Risk Factor Surveillance System Survey (BRFSS) Data Atlanta, Georgia: U.S. Department of Health and Human Services, Centers for Disease Control and Prevention; 1984–2014 https://www.cdc.gov/brfss/index.html.

[25] Wang Y, Sung HY, Lightwood J, Chaffee BW, Yao T, Max W. Health Care Utilization and Expenditures Attributable to Smokeless Tobacco Use Among US Adults. Nicotine Tob Res. 2018;20(11):1359–68. doi: 10.1093/ntr/ntx196 29059335 PMC6154986

[26] Xu X, Shrestha SS, Trivers KF, Neff L, Armour BS, King BA. U.S. healthcare spending attributable to cigarette smoking in 2014. Prev Med. 2021;150:106529. doi: 10.1016/j.ypmed.2021.106529 33771566 PMC10953804

[27] Wang Y, Sung HY, Yao T, Lightwood J, Max W. Health Care Utilization and Expenditures Attributable to Cigar Smoking Among US Adults, 2000–2015. Public Health Rep. 2018;133(3):329–37. doi: 10.1177/0033354918769873 29688130 PMC5958399

[28] Kava CM, Hannon PA, Harris JR. Use of cigarettes and e-cigarettes and dual use among adult employees in the US workplace. Prev Chronic Dis. 2020;17:E16. doi: 10.5888/pcd17.190217 32078502 PMC7085907

[29] Stillman FA, Hartman AM, Graubard BI, Gilpin EA, Murray DM, Gibson JT. Evaluation of the American Stop Smoking Intervention Study (ASSIST): a report of outcomes. J Natl Cancer Inst. 2003;95(22):1681–91. doi: 10.1093/jnci/djg098 14625259

[30] Zhao X, Alexander TN, Hoffman L, Jones C, Delahanty J, Walker M, et al. Youth receptivity to FDA’s The Real Cost Tobacco Prevention Campaign: Evidence from message pretesting. Journal of Health Communication. 2016;21:1153–60. doi: 10.1080/10810730.2016.1233307 27736365 PMC5101168

[31] Kranzler EC, Gibson LA, Hornik RC. Recall of “The Real Cost” anti-smoking campaign is specifically associated with endorsement of campaign-targeted beliefs. Journal of Health Communication. 2017;22:818–28. doi: 10.1080/10810730.2017.1364311 28937865 PMC5822679

[32] Boynton MH, Agans RP, Bowling JM, Brewer NT, Sutfin EL, Goldstein AO, et al. Understanding how perceptions of tobacco constituents and the FDA relate to effective and credible tobacco risk messaging: A national phone survey of U.S. adults, 2014–2015. BMC Public Health. 2016;16(1):516. doi: 10.1186/s12889-016-3151-5 27333921 PMC4918079

[33] Graham AL. Engaging People in Tobacco Prevention and Cessation: Reflecting Back Over 20 Years Since the Master Settlement Agreement. Ann Behav Med. 2020;54(12):932–41. doi: 10.1093/abm/kaaa089 33416838

