# Supplementary material for: Smoking and healthcare expenditure reductions associated with the California Tobacco Control Program, 1989 to 2019: A predictive validation
Source: PLoS One. 2023 Mar 16;18(3):e0263579. doi: 10.1371/journal.pone.0263579 (PMC10019627; doi:10.1371/journal.pone.0263579)
Supplement: S1 Text — (DOCX) [file pone.0263579.s001.docx]

Supplemental Text:

Smoking and Healthcare Expenditure Reductions Associated with the California Tobacco Control Program, 1989 to 2019: A Predictive Validation

James M. Lightwood^1^, Steve Anderson^2, 3^, Stanton A. Glantz^4^

Department of Clinical Pharmacy

School of Pharmacy

University of California, San Francisco

August 26, 2022

1 Department of Clinical Pharmacy, University of California, San Francisco

2 JPMorgan Chase & Co., San Francisco, California, United States of America

3 Analytical Steve Consulting, San Francisco, California, United States of America

^4^Center for Tobacco Control Research and Education, Philip R. Lee Institute for Health Policy Studies, and Department of Medicine, University of California San Francisco

Supported by California Department of Health and Human Services contract CTCP 19-10620 National Institute on Drug Abuse grant DA-043950. The funding agencies played no role in the conduct of the research or preparation of the manuscript.

Table of Contents

[Technical Appendix 3](#_Toc112429646)

[Data 3](#_Toc112429647)

[Methods 3](#_Toc112429648)

[Published model 3](#_Toc112429649)

[Model specification with measurement adjustment forbreak in BRFSS survey methodology in 2011 and following years. 4](#_Toc112429650)

[Details on statistical methods 5](#_Toc112429651)

[Sensitivity analysis and Details on Diagnostic Test for Weak Instruments 8](#_Toc112429652)

[Results 12](#_Toc112429653)

[Notes on Figures for Details of Results 15](#_Toc112429654)

[Discussion: Calculations for other estimates from literature 31](#_Toc112429655)

[Data Appendix 33](#_Toc112429656)

[REFERENCES 42](#_Toc112429657)

# Technical Appendix

## Data

All data used in ECTCP [1] were still available, except for sub-state price indices used to calculate the California state price index [2]; alternative consistent price index data for California were obtained from the California Department of Finance. [3]

The regional price indices used the East, South and Midwestern Regions. The California price index from the California Department of Finance did not have categories of all-items less Medical Care. Therefore, we used the all-item price index; it also did not have Medical Care Services, so we used Medical Care. An adjusted West Region index which was derived from the population weighted West Region index after subtracting out the population weighted California Index using the same methods as in ECTCP [1].

## Methods

### Published model

Current adult smoking prevalence in California:

$\left( {prev}_{c,t}-{prev}_{CA,t} \right)={\alpha_{0}+\alpha}_{1}\left( {EC}_{CA,t-1}-{EC}_{c,t-1} \right)+\alpha_{2}\left( p_{CA,t-1}-p_{c,t-1} \right)+\alpha_{3}\left( y_{CA,t-1}-y_{c,t-1} \right)+\varepsilon_{1a,t}$ (S1a)

Cigarette Consumption per Smoker:

$\left( {cpsa}_{c,t}-{cpsa}_{CA,t} \right)=\beta_{0}+\beta_{1}\left( {EC}_{CA,t-1}-{EC}_{c,t-1} \right)+\beta_{2}\left( p_{CA,t-1}-p_{c,t-1} \right)+\beta_{3}\left( y_{CA,t-1}-y_{c,t-1} \right)+\varepsilon_{2a,t}$ (S2a)

NIPA Healthcare Expenditures:

$n_{CA,t}=\gamma_{0}+\gamma_{1}n_{c,t}+\gamma_{2}\left( {prev}_{c,t-1}-{prev}_{CA,t-1} \right)+\gamma_{3}\left( {cpsa}_{c,t-1}-{cpsa}_{CA,t-1} \right)+\gamma_{4}\left( y_{CA,t-1}-y_{c,t-1} \right)+\varepsilon_{3a,t}$

(S3a)

CMS Healthcare Expenditures:

$h_{CA,t}=\delta_{0}+\delta_{1}h_{c,t}+\delta_{2}\left( {prev}_{c,t-1}-{prev}_{CA,t-1} \right)+\delta_{3}\left( {cpsa}_{c,t-1}-{cpsa}_{CA,t-1} \right)+\delta_{4}\left( y_{CA,t-1}-y_{c,t-1} \right)+\varepsilon_{4a,t}$

(S4a)

### Model specification with measurement adjustment forbreak in BRFSS survey methodology in 2011 and following years.

Published Model

Current adult smoking prevalence in California:

$\left( {prev}_{c,t}-{prev}_{CA,t} \right)={\alpha_{0}+\alpha}_{1}\left( {EC}_{CA,t-1}-{EC}_{c,t-1} \right)+\alpha_{2}\left( p_{CA,t-1}-p_{c,t-1} \right)+\alpha_{3}\left( y_{CA,t-1}-y_{c,t-1} \right)+\alpha_{4}D2011+\alpha_{5}D2011*\left( {prev}_{c,t}-{prev}_{CA,t} \right)+\varepsilon_{1b,t}$ (S1b)

Cigarette Consumption per Smoker:

$\left( {cpsa}_{c,t}-{cpsa}_{CA,t} \right)=\beta_{0}+\beta_{1}\left( {EC}_{CA,t-1}-{EC}_{c,t-1} \right)+\beta_{2}\left( p_{CA,t-1}-p_{c,t-1} \right)+\beta_{3}\left( y_{CA,t-1}-y_{c,t-1} \right)+\beta_{4}D2011+\beta_{5}D2011*\left( {cpsa}_{c,t}-{cpsa}_{CA,t} \right)+\varepsilon_{2b,t}$ (S2b)

NIPA Healthcare Expenditures:

$n_{CA,t}=\gamma_{0}+\gamma_{1}n_{c,t}+\gamma_{2}\left( {prev}_{c,t-1}-{prev}_{CA,t-1} \right)+\gamma_{3}\left( {cpsa}_{c,t-1}-{cpsa}_{CA,t-1} \right)+\gamma_{4}\left( y_{CA,t-1}-y_{c,t-1} \right)+\gamma_{5}D2011*\left( {prev}_{c,t-1}-{prev}_{CA,t-1} \right)+\gamma_{6}D2011*\left( {cpsa}_{c,t-1}-{cpsa}_{CA,t-1} \right)+ \varepsilon_{3b,t}$ (S3b)

CMS Healthcare Expenditures:

$h_{CA,t}=\delta_{0}+\delta_{1}h_{c,t}+\delta_{2}\left( {prev}_{c,t-1}-{prev}_{CA,t-1} \right)+\delta_{3}\left( {cpsa}_{c,t-1}-{cpsa}_{CA,t-1} \right)+\delta_{4}\left( y_{CA,t-1}-y_{c,t-1} \right)+\delta_{5}D2011*\left( {prev}_{c,t-1}-{prev}_{CA,t-1} \right)+\delta_{6}D2011*\left( {cpsa}_{c,t-1}-{cpsa}_{CA,t-1} \right)+\varepsilon_{4b,t}$ (S4b)

Forecast Model (corresponding to equations 1-4 in main text)

Current adult smoking prevalence in California:

$\left( {prev}_{c,t-1}-{prev}_{CA,t} \right)=\alpha_{0}+\alpha_{1}\left( {EC}_{CA,t-1}-{EC}_{c,t-1} \right)+\alpha_{2}\left( p_{CA,t-1}-p_{c,t-1} \right)+\alpha_{3}\left( y_{CA,t-1}-y_{c,t-1} \right)+\alpha_{4}D2011+\alpha_{5}D2011*\left( {prev}_{c,t-2}-{prev}_{CA,t-1} \right)+\varepsilon_{1c,t}$ (S1c)

Cigarette Consumption per Smoker:

$\left( {cpsa}_{c,t-1}-{cpsa}_{CA,t} \right)=\beta_{0}+\beta_{1}\left( {EC}_{CA,t-1}-{EC}_{c,t-1} \right)+\beta_{2}\left( p_{CA,t-1}-p_{c,t-1} \right)+\beta_{3}\left( y_{CA,t-1}-y_{c,t-1} \right)+\beta_{4}D2011+\beta_{5}D2011*\left( {cpsa}_{c,t-2}-{cpsa}_{CA,t-1} \right)+\varepsilon_{2c,t}$ (S2c)

NIPA Healthcare Expenditures:

$n_{CA,t}=\gamma_{0}+\gamma_{1}n_{c,t-1}+\gamma_{2}\left( {prev}_{c,t-2}-{prev}_{c,t-1} \right)+\gamma_{3}\left( {cpsa}_{c,t-2}-{cpsa}_{CA,t-1} \right)+\gamma_{4}\left( y_{CA,t-1}-y_{c,t-1} \right)+\gamma_{5}D2011*\left( {prev}_{c,t-2}-{prev}_{CA,t-1} \right)+\gamma_{6}D2011*\left( {cpsa}_{c,t-2}-{cpsa}_{CA,t-1} \right)+\varepsilon_{3c,t}$ (S3 c)

CMS Healthcare Expenditures:

$h_{CA,t}=\delta_{0}+\delta_{1}h_{c,t-1}+\delta_{2}\left( {prev}_{c,t-2}-{prev}_{CA,t-1} \right)+\delta_{3}\left( {cpsa}_{c,t-2}-{cpsa}_{CA,t-1} \right)+\delta_{4}\left( y_{CA,t-1}-y_{c,t-1} \right)+\delta_{5}D2011*\left( {prev}_{c,t-2}-{prev}_{CA,t-1} \right)+\delta_{6}D2011*\left( {cpsa}_{c,t-2}-{cpsa}_{CA,t-1} \right)+\varepsilon_{4c,t}$ (S4 c)

Where $D2011$ is a dummy variable equal to 0 until year 2010 and 1 for the following years.

### Details on statistical methods

**Regression methods and diagnostic statistics**

We used standard time series regression techniques appropriate for non-stationary variables. Under the assumption cointegrating relationship involving variables that have unit root, or random walk, nonstationarity, the appropriate procedure to determine any long run relationship between the variables is to estimate equations 1 to 4 in the main text (or S1a to S4c above, when adding the measurement model) as static regressions with no lagged variables. Cointegrating relationships estimated with lagged explanatory variables estimate the combined long-run relationship with short-run dynamics. Ordinary least squares estimates of cointegrating regressions may poorly approximate t-distributions and be biased in small samples, so irrelevant instrumental variables (IIV) estimates [4, 5] were used for estimation. We use orthogonal basis function IIV regression methods developed by Phillips [5] that are valid asymptotically, and with evidence of good performance in small samples.

Several diagnostic tests were conducted to evaluate regression specifications and assumptions. Parameter stability was assessed using Cumulative Sum (CUSUM) tests on recursive and ordinary residuals of the regression, using ordinary least squares regression residuals. The stability of the regression coefficients was assessed using recursive regression estimates. Multicollinearity in the explanatory variables was assessed using Variance Inflation Factors (VIFs). Heteroskedasticity was assessed using the Cook-Weisberg test. The presence of a unit root in the residuals was assessed using the Phillips-Perron unit root test. Bartlett’s White Noise Test was used to test for independent identically distributed (iid) residuals. Autocorrelation statistics were calculated for the residuals. The presence of potentially influential observations was assessed using leverage versus squared residuals plots. The strength of the irrelevant instrumental estimates were evaluated using the Montiel-Pflueger robust weak instrument tests [6]. The five percent significance level was used for each test.

The original and updated time series were compared using pairwise correlation coefficients. The Root Mean Square Error (RMSE) of model predictions of each equation was used as the primary criterion to evaluate model and estimate prediction adequacy. The correlation coefficient was used as the secondary criterion. The relative RMSE was also calculated. The Root Mean Square Model Error (RMSME) was also calculated for the NIPA and CMS healthcare expenditure. The RMSME was estimated for the health care equation predictions using the predicted time series for smoking prevalence and cigarette consumption per smoker used as explanatory variables. The RMSME was the more important criterion for estimation of CTCP program effect because health expenditure estimates that use predicted prevalence and consumption are needed to evaluate the observed time series versus the counterfactual of no CTCP program. The RMSME for the NIPA measure of health care expenditure was considered the most important criterion because the NIPA estimates are stable over successive revisions, and therefore more consistent than the CMS measure.

Adjustment of an IIV estimator for the long run covariance matrix of the Brownian motions of the regression variables (which is achieved by including leading first differences of explanatory variables in the IIV regressions) was not used for the estimates because they involve leading variables which cannot be used for prediction and forecasting. Inclusion of the leading first differences did not noticeably change the regression estimates. Sinusoidal polynomial basis functions were used for IIV instruments as suggested in Phillips [5] and twenty instruments, following Phillips’ recommendation for determining the optimal number of instrumental variables. Sensitivity analysis indicated that results were insensitive to alternative choice for basis functions and number of instruments [5]. Two stage least squares instrumental regressions were estimated using Stata command *reg3* and *ivregress*. Robust regression estimates and estimates with variance-covariance matrices robust to autocorrelation used the Stata procedures *rreg* and *newey*, and sandwich (HAC) variance estimators (for *reg3*) respectively

The following Stata commands were used for the regression diagnostics: CUSUM test for parameter stability: *estat sbcusum*, heteroskedasticity test: *estat hettest*, Bartlett’s White Noise test: *wntestb*, autoocorrelation coefficients: *corrgram*, potentially influential observations*: lvr2plot*. The weak instrument tests used the Stata add-in *weakivtest* [7].

**Descriptive statistics for time series properties and tests for stationarity**

Tests for stationarity are the same as in the previous research [8]. The Phillips-Perron unit root test (Stata command pperon) was used to test for unit roots, using level or trend nonstationarity as the null [9]. (A unit root tests whether a variable y evolves as a random walk, i.e., with the dynamic relation y_t_ = y_t-1_ + constant + deterministic trend + error_t_.) The KPSS test [10], which uses level or trend stationarity as the null, was used to confirm the results of the Phillips-Perron test and to check for sensitivity or results to choice of null hypothesis. The Phillips-Peron test may have low power in small samples [9], so the KPSS test and analysis of autocorrelation function (ACF) and partial autocorrelation function (PCAFs) were used to evaluate stationarity when there was a conflict and Phillips-Perron showed borderline significant results. Automatic lag order selection was used for unit root tests and ACF/PACF analysis: ceiling(4[N/100]^2/9^) for Phillips-Perron; Hobijn [11] automatic bandwidth selection for KPSS test; min(ceiling [N/2-1], 40) for ACF/PACF.

Data processing used the SAS version 9.4 [12] and R [13]. Statistical analysis used Stata version 16 [14]. Simulations used the Yasai excel add-in [15].

### Sensitivity analysis and Details on Diagnostic Test for Weak Instruments

Inclusion of additional explanatory variables in all equations

The following variables were included individually in all equations: state level prevalence of Black, Hispanic, elderly, older adult male (age 45-64), older adult women (age 45-64), women of child-bearing age, young adults and adults (age 18 to 45); poverty rate; unemployment rate; prevalence of two measures of educational attainment (high school, and post high school college/technical degree at age 25); and the proportion of the population enrolled in Medicaid. In order to check whether there was an effect of a group of correlated variables, we also did a principal components analysis on the covariance matrix of these variables and added the significant principal components (PCs), determined using the scree plot, to the regressions. A measure of health care supply shifts, per capita hospital beds, was added to the regressions for health care (Eqs. 3 and 4).

Choice of control States

The study design of this research differs from Abadie [16] with regard to the choice of control states. That study constructs a synthetic control population that is judged similar to California (the intervention group), and takes difference between per capita cigarette consumption between California and control population to estimate program effect. That approach is similar to use of matching for statistical adjustment, and requires that all of the control states have no tobacco program. Our study uses regression-based estimates assuming nonstationary time series and therefore requires that the weaker assumption that intervention and control time series for tobacco control expenditure, prevalence of smoking and mean consumption per smoker not be cointegrated. A disadvantage of the Abadie approach is that it cannot be carried forward in time if suitable control states no longer exist because they subsequently started substantial tobacco control programs. The results of our study should be less sensitive to the choice of control state populations, except that the time series of the control and intervention populations cannot be cointegrated and must have enough independent variation to achieve sufficient power.

The choice of control states used in this research was the same as in ECTCP, which were based on an analysis of state level tobacco control expenditure to the year 2000, where states that had no substantial tobacco control expenditure or continuous control program. These controls were chosen for research on the construction of synthetic control groups for program evaluation [16]; that research estimated program effects using simple differences in measures of smoking behavior between California (the intervention group) and expenditures of a control group modeled from the 38 control states. Therefore, those results are sensitive to the choice of the control group and are dependent on those states having essentially no program at all. This research uses regression estimates, which are much less sensitive to the choice of control states, and the different distinct time paths of California and control state tobacco control expenditure are more important. However, the control states’ tobacco control expenditures and control programs could have changed substantially since 2000. Therefore, a sensitivity analysis was conducted to compare the regression estimates for the effect of state tobacco control expenditure on prevalence and cigarette consumption for different groups of control states with relatively homogeneous levels of tobacco control expenditure.

This sensitivity analysis was conducted as follows. After examination of the data, the total cumulative real per capita state tobacco control expenditure at the end of the sample was a good index of the average tobacco control expenditure over the sample for the fifty states and District of Columbia (DC). Using this index, the states and DC were divided into tertiles. The equations for smoking prevalence ((Eq. 1) and cigarette consumption (Eq. 2) in the main text were re-estimated using each tertile as the control group. The regression coefficients were compared to the 38 controls used for the main analysis and the recursive estimates were checked for stability.

Discount Rate for Tobacco Control Expenditure

ECTCP assumed that there was no decay in the effect of tobacco control expenditures over time in the regressions predicting smoking prevalence and mean cigarette consumption (Eqs. 1 and 2); that is their effect had perfect memory, or a discount rate of zero (that is., the times series of real per capita tobacco control spending that affective smoking behavior is modeled as a unit-root nonstationary process). As the time series sample period increases, this assumption should be examined. Any discounting in the effect of tobacco control spending will result in a stationary time series, which is incompatible asymptotically with a cointegrating regression analysis. However, in a relatively short time series, using cointegration analysis may be the best approach to regression modelling if any discounting is close to zero [17]. Therefore, this sensitivity analysis explores the robustness of the analysis as an adequate approximation to a short time series that has an unknown order of integration, but is likely very close to unit-root nonstationary.

The first step was to test for plausible stationary time series processes as alternatives to unit root stationarity, which was assumed in the main analysis. The two candidates are a stationary long memory process and a stationary exponential decay process. Estimation using the Autoregressive Fractional Integrated Moving Average (ARFIMA) process was used to distinguish between these two possibilities [18].

A grid search was conducted over discount rates for California and control state tobacco control expenditure for discount rates of zero (perfect memory and unit root non-stationary) to 100 percent per year. Eqs. 1 and 2 were re-estimated for those discount rates. The RMSE of the model predictions and other regression diagnostics for the regressions using each discount rate were compared. The effect of the CTCP program was re-estimated for the lowest plausible bound for the discount rate to determine the effect on the results.

If the annual discount rate for control expenditures is greater than zero, then it is no longer unit-root non-stationary, and cointegration analysis is approximate in most respects. Serious distortion can occur in the size of tests for stationarity and of coefficient restrictions. However, these problems are resolved if the regressors are exogenous, or valid instrumental variable estimators are used, as they are in this case [19].

Exploratory analysis of the implications of the introduction of e-cigarettes

Estimates of trends in e-cigarette use were calculated for California and the control states and included in the Eqs. 1 to 4 of the final forecast model. The model was re-estimated to determine possible effects of including the growing prevalence of e-cigarettes in the analysis.

Diagnostic tests for weak instruments

The results of the diagnostic tests for weak instruments are reported with estimates of maximum weak instrument bias in the estimated coefficients.

## Results

Effect of including additional explanatory variables in all equations.

We only found two cases where adding the variable, or PCs, made a statistically significant change to the coefficients of the variables of policy interest in the model (tobacco control expenditure in equations 1 and 2; prevalence of smoking and mean consumption per smoker in equations 3 and 4).

The addition of a principal component that described increased Black, Hispanic population and poverty rate, and decreased Medicaid enrollment in California compared to the control states changed the coefficient (*α*_1_) for prevalence (Eq. 1) from 0.0503 (SE 0.0107) to 0.0848 (SE 0.0366) (P for difference = 0.37). The coefficient *δ*_2_ in equation 4 for effect or mean consumption per smoker on the CMS measure of healthcare expenditure changed from -5.00 (SE 0.573) to -3.12 (SE 0.614) (P for difference 0.025) when the poverty rate was added the regression.

Given the dozens of equations that were estimated, neither of these results would have survived even more powerful post hoc adjustments for multiple tests. So, these results could well be by chance. In fact, we believe that using predictive models to evaluate out-of-sample performance of model specifications is useful for just this situation, which cannot be addressed adequately using in-sample inferential tests.

Choice of Control State Populations

The time paths of the control state tertiles of the control states as ranked by the index of total cumulative program expenditure at the end of the sample are shown in Figure S5. The time series of tobacco control expenditure, smoking behavior and health care expenditure for the control population for each tertile were not cointegrated with the California series. Therefore, in a nonstationary unit-root system, they should allow unbiased regression estimates in a cointegrating regression. The coefficient estimates of the model were not statistically significantly different from the estimates for forecast model at the 5% level for each of the lower two tertiles of the control states. The estimates for highest tertile were significantly different, but the results of the regression results were poor, judged by regression diagnostics and predictive performance. The time paths of tobacco control spending by control states in the highest tertile control states were very close to that of California after 2008, and therefore the power to estimate a difference in the dependent variables between California and this group of control states may be low. Use of the original 38 state control population does not substantially change the results for results contrasting California with populations that have large differences in per capita tobacco control expenditure (Table S4).

Discount Rate for Tobacco Control Expenditure

No evidence was found for a stationary long-memory component in the time series for smoking prevalence or mean cigarette consumption; therefore, only exponential discounting was examined.

Eq. 1 and Eq. 2 of the forecast model were re-estimated using an annual discount rate of 0.025 to 1. The RMSE of model predictions rose rapidly for discount rates above 2.5 percent per year for California smoking prevalence (Eq. 1) and started rising for rates above 5 to 7.5 percent per year for cigarette consumption (Eq. 2) (Figure S6). The RMSE was unstable for consumption when the annual discount rate for the control states were increased above 50 percent; regression diagnostics start to display problems such as instability and autocorrelation in the regression residuals. With higher discount rates, the estimated regression coefficients for tobacco control expenditure for tobacco expenditure, and resulting health care expenditure, increased by 6 percent to 100 percent. Therefore, the base case of not discounting produces lower, more conservative, estimates of CTCP program effect.

Exploratory analysis of the implications of the introduction of e-cigarettes

We used available data (three to four annual observations for each state combined with three national observations to establish early trends) on prevalence of adult e-cigarette use in the US since 2007 to calculate a very rough estimate of the trends in California and control state prevalence. [20-22] We entered these trends into the cigarette prevalence and consumption per smoker regressions (Eqs. 1 and 2), and the health care expenditure regressions (Eqs. 3 and 4). Prevalence of e-cigarette use was higher in the control states than California, perhaps due to lower overall tobacco consumption in California. Several specifications were used. The regression coefficients for e-cigarette prevalence never approached statistical significance (P > 0.4 for all regressions). The coefficients for tobacco control expenditure in Eqs 1 and 2, and for prevalence and consumption in Eqs 3 and 4 remained statistically significant and the changes compared to the estimates based only on cigarettes were not statistically (P for difference > 0.59). Thus, the currently identifiably effect of omitting e-cigarette use on the results is currently negligible. Because the number of observations on state level prevalence of e-cigarette use will be very small for a number of years, other methods such dynamic panel estimators for nonstationary data will be needed for informative estimates of the effect of e-cigarette (and other new product) use on reliable forecasting models.

The changes in the point estimates of the coefficients are described below.

Tobacco control expenditure

Prevalence (Eq 1) coefficient increases from 0.0503 to 0.0540 (7% change)

Consumption (Eq 2) coefficient increases from 2.23 to 2.33 (4% change)

NIPA health care expenditure (Eq 3)

Prevalence coefficient increases from -54.5 to -52.3 (4% change)

Consumption coefficient increases from -3.40 to -3.29 (3% change)

CMS health care expenditure (Eq 3)

Prevalence coefficient decreases from -86.3 to -90.6 (5% change)

Consumption coefficient decreases from -5.00 to -5.24 (5% change)

Inclusion of e-cigarettes in the model increases point estimate for effectiveness of tobacco control expenditure in reducing use of combustible cigarettes. The effect of tobacco control expenditure on combustible cigarette use has ambiguous effects on the point estimates for per capita health care expenditure. These changes, however, do not even approach statistical significance, so the most reasonable interpretation is that including e-cigarettes in the model does not detectably affect the estimates of the effects of the California Tobacco Control Program.

In conclusion, the effect of omitting e-cigarette use on the results is currently negligible. Because the number of observations on state level prevalence of e-cigarette use will be very small for a number of years, other methods such as dynamic panel estimators for nonstationary data will be needed for informative estimates of the effect of e-cigarette and ENDS use for future development of reliable forecasting models that include the effects of new products.

Diagnostic tests for weak instruments

The null hypothesis that the instruments are weak is rejected at the 5 percent significant level for reasonable levels of worst-case bias. The null hypothesis of weak instruments was rejected for the worst-case relative bias of less than five percent for the coefficients of all the explanatory variables except for the following.

1. consumption in the CMS healthcare expenditure (Eq. 4): approximately 7 percent

2. prevalence in the NIPA expenditure (Eq. 3): approximately 12 percent

3. prevalence in the CMS expenditure (Eq. 4): approximately 18 percent

## Notes on Figures for Details of Results

Panels a and b in Figure S1 show the recursive regression coefficients of the forecast model for tobacco control expenditure on prevalence (Eq. 1) and mean consumption (Eq.2) Panels c and d show the recursive regression coefficients for the effect of prevalence and mean consumption on NIPA health care expenditures, respectively (Eq. 3). Panels e and f show the recursive regression coefficients for the effect of prevalence and mean consumption on CMS health care expenditures, respectively (Eq. 4). Note that there are sign changes in some coefficients do to different (but equivalent) regression specifications used for the recursive regressions.

Figures S2 and S3 show the observed and predicted prevalence of smoking and mean cigarette consumption per smoker for California and the difference between California and the control states. Figure S4 shows the NIPA, and CMS per capita health care expenditures for California. There are two predictions for NIPA and CMS per capita health care expenditures. One prediction (black lines in Figure S4) is for the regressions that use the observed prevalence of smoking and mean consumption per smoker as explanatory variables. The dashed gray lines in Figure S4 are the predictions that use the predicted prevalence of smoking (from Eq. 1) and mean consumption per smoker (from Eq. 2). This second set of predictions is needed to estimate program effects, where the model predictions of the observed annual prevalence and mean consumption over time) must be compared to the counterfactual prevalence and mean consumption with tobacco education expenditures set to zero for the counterfactual of no CTCP. The predictions of the estimated models with updated data track the observed data closely. Only one to two observations fall outside the 95 percent forecast interval for individual observations for any of the predicted time series, as expected with the respective sample sizes.

The model predictions (Figures S2, S3, and S4) fit the data well, except for the year 2011. The discontinuities at 2011 are due to the break in the BRFSS survey methodology in that year. The forecast model predictions with lagged explanatory variables, but also use lagged variables that adjust for the existence of the break, so cannot completely eliminate the break in the model predictions of the observed data series. The regression estimates of the counterfactual case of no CTCP expenditures are shown in Figures S2 to S4 (solid gray lines) for prevalence of smoking, mean cigarette consumption, NIPA and CMS health care expenditures.

Table S1. Estimated California smoking prevalence, cigarettes per capita, and per capita healthcare expenditures, model validation and updated estimates (numbers in parenthesis are standard errors).

| Equation | Dependent Variable | Statistic | ECTPC, 2013 | ECTCP, 'published', updated data to 2008 | ECTCP, 'published', updated data | ECTCP, 'forecast', updated data | Dimension |
| --- | --- | --- | --- | --- | --- | --- | --- |
| Model Equations | |  |  |  |  |  |  |
| 1 | (*prev_c, t_ – prev_CA, t_*) | *α0* | 6.30 (0.610) | 6.54 (1.37) | 5.95 (1.20) | 6.29 (1.29) |  |
|  | (*prev_c, t-1_ – prev_CA, t_*)^**^ | *α1* | 0.0497 (0.00347) | 0.0489 (0.0110) | 0.0494 (0.00983) | 0.0503 (0.0107) | /$ per capita |
|  |  | *α2* | 1.00 (0.477) | 0.862 (0.705) | 0.940 (0.571) | 0.419 (0.641) | /$ per pack |
|  |  | *α3* | -0.416 (0.0730) | -0.432 (0.175) | -0.352 (0.155) | -0.312 (0.163) | /$1000 per capita |
|  |  | *R^2^* (%) | 77 | 76 | 79 | 73 |  |
|  |  | *r1* | 0.154 | 0.134 | 0.156 | -0.0230 |  |
| 2 | (*cpsa_c, t_ – cpsa_CA, t_*) | *β0* | 67.9 (10.2) | 92.8 (33.6) | 55.8 (31.2) | 52.5 (36.9) |  |
|  | (*cpsa_c, t-1_ – cpsa_CA, t_*)^**^ | *β1* | 1.39 (0.132) | 2.11 (0.271) | 2.02 (0.267) | 2.23 (0.303) | /$ per capita |
|  |  | *β2* | 26.6 (6.80) | 24.2 (17.3) | 37.7 (16.1) | 41.0 (17.9) | /$ per pack |
|  |  | *β3* | -2.97 (1.21) | -2.15 (4.30) | 2.55 (3.93) | 3.02 (4.69) | /$1000 per capita |
|  |  | *R^2^* (%) | 81 | 82 | 79 | 78 |  |
|  |  | *r1* | 0.148 | 0.121 | 0.190 | -0.0886 |  |
| 3 | *n_CA, t_* | *γ0* | 550 (433) | 536 (404) | 434 (297) | 351 (397) | $ |
|  |  | *γ1* | 1.15 (0.180) | 0.994 (0.164) | 1.06 (0.114) | 1.02 (0.142) |  |
|  |  | *γ2* | -35.4 (9.85) | -64.3 (16.1) | -69.5 (13.8) | -54.5 (16.7) | $/%point |
|  |  | *γ3* | -3.14 (0.786) | -3.16 (0.398) | -3.22 (0.335) | -3.40 (0.433) | $/pack per smoker |
|  |  | *γ4* | 108 (6.79) | 69.1 (15.7) | 62.3 (12.1) | 88.6 (15.9) | $/$1000 per capita |
|  |  | *R^2^* (%) | 80 | 90 | 92 | 86 |  |
|  |  | *r1* | 0.262 | 0.389 | 0.415* | -0.00420 |  |
| 4 | *h_CA, t_* | *δ0* | 1056 (112) | 1481 (373) | 1627 (303) | 1477 (279) | $ |
|  |  | *δ1* | 0.847 (0.0542) | 0.980 (0.0988) | 0.908 (0.0590) | 0.845 (0.0492) |  |
|  |  | *δ2* | -67.8 (7.31) | -149 (32.0) | -130 (25.3) | -86.3 (21.5) | $/%point |
|  |  | *δ3* | -5.48 (0.928) | -5.59 (0.865) | -5.24 (0.641) | -5.00 (0.573) | $/pack per smoker |
|  |  | *δ4* | 107 (22.3) | 41.1 (30.8) | 59.7 (23.0) | 110 (20.5) | $/$1000 per capita |
|  |  | *R^ ^* (%) | 89 | 87 | 93 | 94 |  |
|  |  | *r1* | 0.486* | 0.506* | 0.499* | 0.461* |  |

^*^significant at the 5% level.

^**^ specification of dependent variable for the ‘forecast’ model.

*r_1_*: first order autocorrelation coefficient.

*prev_j, t_*: Prevalence of current smoking in population j, for California and control states in year t,(percentage points).

*cps_j, t_*: Cigarettes consumption per current smoker in population j, for California and control states in year t, (packs/year per smoker).

*EC_j, t_*: Cumulative per capita funding in population j, for California and control states in year t, (dollars).

*p_j, t_*: Price per pack of cigarettes in population j, for California and control states in year t, (dollars).

*y_j, t_*: Per capita personal income in population j, for California and control states in year t, (thousands of dollars).

*n_j, t_*: Per capita healthcare expenditures in population j, for California and control states in year t, (thousands of dollars).

*h_j, t_*: Per capita healthcare expenditures in population j, for California and control states in year t, (thousands of dollars).

Note: dollar amounts are in 2010 dollars.

Table S2 -Root Mean Square Error and Root Means Square Model Error of predicted versus observed

| Dependent Variable to be predicted | Root Mean Square Error of Predictions | | |
| --- | --- | --- | --- |
|  | ECTCP estimates applied to updated data to most recent data available | ‘Published’ Model  with updated data to most recent data available | ‘Forecast’ model with updated data to most recent data available |
| Root Mean Square Error |  |  |  |
| Prevalence (s), control - CA | 0.904 | 0.737 | 0.852 |
|  |  |  |  |
| Consumption (cpsa), control - CA | 47.3 | 21.3 | 23.7 |
|  |  |  |  |
| Per capita real NIPA healthcare expenditure, CA |  |  |  |
| with observed s and cpsa | 159 | 61.5 | 79.3 |
| Per capita real CMS healthcare expenditure, CA |  |  |  |
| with observed s and cpsa | 204 | 104 | 92.9 |
| Root Mean Square Model Error |  |  |  |
| Per capita real NIPA healthcare expenditure, CA |  |  |  |
| with predicted s and cpsa | -- | 84.6 | 79.3 |
| Per capita real CMS healthcare expenditure, CA |  |  |  |
| with predicted s and cpsa | -- | 123 | 130 |

Table S3. Correlations of predicted versus observed

| Dependent Variable to be predicted | Correlations | | | |
| --- | --- | --- | --- | --- |
|  | ECTCP estimates applied to old data used in 2013 publication | 2013 estimates applied to updated data to most recent data available | Re-estimated 2013 model with updated data to most recent data available | Estimated ‘forecast’ version 2013 model with updated data to most recent data available |
| Prevalence (s), control - CA | 0.875 | 0.846 | 0.875 | 0.874 |
|  |  |  |  |  |
| Consumption (cpsa), control - CA | 0.891 | 0.871 | 0.891 | 0.892 |
|  |  |  |  |  |
| Per capita real NIPA healthcare expenditure, CA |  |  |  |  |
| with observed s and cpsa | 0.816 | 0.870 | 0.957 | 0.927 |
|  |  |  |  |  |
| Per capita real CMS healthcare expenditure, CA |  |  |  |  |
| with observed s and cpsa | -- | 0.970 | 0.950 | 0.975 |
| Root Mean Square Model Error |  |  |  |  |
| Per capita real NIPA healthcare expenditure, CA |  |  |  |  |
| with predicted s and cpsa | -- | -- | 0.543 | 0.552 |
| Per capita real CMS healthcare expenditure, CA |  |  |  |  |
| with predicted s and cpsa | -- | -- | 0.958 | 0.953 |

Table S4. Estimated California smoking prevalence, cigarettes per capita, health care expenditures, by tertile of control state expenditure.

| Equation | Dependent Variable | Statistic | coefficient | low 95% CI | high 95% CI | P-value for difference from base case |
| --- | --- | --- | --- | --- | --- | --- |
| Model Equations | |  |  |  |  |  |
| 1 | (*prev_c, t_ – prev_CA, t_*) | *α1 (EC)* |  |  |  |  |
|  | (*prev_c, t-1_ – prev_CA, t_*)** | low tertile | 0.0319 | 0.0151 | 0.0487 | 0.159 |
|  |  | medium tertile | 0.0462 | 0.0251 | 0.0675 | 0.167 |
|  |  | high tertile | -0.0687 | -0.0687 | 0.0294 | 0.020 |
| 2 | (*cps_c, t_ – cps_CA, t_*) | *β1 (EC)* |  |  |  |  |
|  | (*cps_c, t-1_ – cps_CA, t_*)** | low tertile | 1.44 | 0.886 | 1.99 | 0.0661 |
|  |  | medium tertile | 2.08 | 1.58 | 2.58 | 0.836 |
|  |  | high tertile | -2.57 | -7.01 | 1.87 | 0.0380 |
| 3 | *n_CA, t_* | *γ2 (prev)* |  |  |  |  |
|  |  | low tertile | -73.3 | -110 | -35.8 | 0.507 |
|  |  | medium tertile | -55.0 | -117 | 7.64 | 0.963 |
|  |  | high tertile | -17.0 | -92.3 | 58.3 | 0.422 |
|  |  | *γ3 (cpsa)* |  |  |  |  |
|  |  | low tertile | -3.42 | -4.49 | -2.34 | 0.566 |
|  |  | medium tertile | -2.89 | -4.19 | -1.60 | 0.952 |
|  |  | high tertile | -2.39 | -4.48 | -0.297 | 0.648 |
| 4 | *h_CA, t_* | *δ1 (prev)* |  |  |  |  |
|  |  | low tertile | -106 | -168 | -43.8 | 0.636 |
|  |  | medium tertile | -81.5 | -121 | -42.5 | 0.835 |
|  |  | high tertile | 66.0 | 6.25 | 126 | < 0.001 |
|  |  | *δ2 (cpsa)* |  |  |  |  |
|  |  | low tertile | -4.70 | -6.31 | -3.10 | 0.674 |
|  |  | medium tertile | -3.65 | -63.4 | 56.1 | 0.984 |
|  |  | high tertile | -2.15 | -3.58 | -0.712 | 0.031 |

^**^ specification of dependent variable for the ‘forecast’ model.

*prev_j, t_*: Prevalence of current smoking in population j, for California and control states in year t,(percentage points).

*cps_j, t_*: Cigarettes consumption per current smoker in population j, for California and control states in year t, (packs/year per smoker).

*EC_j, t_*: Cumulative per capita funding in population j, for California and control states in year t, (dollars).

(thousands of dollars).

*h_j, t_*: Per capita healthcare expenditures in population j, for California and control states in year t, (thousands of dollars).

Panel a

Panel b

Panel c

Panel d

Panel e

Panel f

Figure S1.- Recursive regression coefficient estimates for variables used to estimate CTCP effect. The years on the horizontal axis indicate the last year in the successive recursive samples.

Panel a.

Panel b.

Figure S2. – Predictions for prevalence of adult current smoking (Eq. 1c). Panel a: control states – California; Panel b: California

Black circles: observed time series; Solid black line: model prediction; Dashed black lines: 95 percent confidence interval for prediction; Dotted black lines: 95 percent forecast interval for individual observations; Solid gray line: model prediction for hypothetical of no CTCP.

Panel a.

Panel b.

Figure S3. – Predictions for mean cigarette consumption per current smoker (Eq. 2c). Panel a: control states – California; Panel b: California

Black circles: observed time series; Solid black line: model prediction; Dashed black lines: 95 percent confidence interval for prediction; Dotted black lines: 95 percent forecast interval for individual observations; Solid gray line: model prediction for hypothetical of no CTCP.

Panel a

Panel b

Figure S4. – Predictions for real per capita health care expenditure, California. Panel a: NIPA measure, (Eq. 3c), Panel b: CMS measure (Eq. 4c)

Black circles: observed time series; Solid black line: model prediction using observed smoking prevalence and mean consumption; Dashed gray line: model prediction using predicted smoking prevalence and mean consumption; Dashed black lines: 95 percent confidence interval for prediction; Dotted black lines: 95 percent forecast interval for individual observations; Solid gray line: model prediction for hypothetical of no CTCP.

Note: vertical axes are real 2019 dollars

Panel a. Panel b.

Panel c.

Figure S5.- Time paths of real per capita expenditure for California (thick black line) and control states (colored lines) by tertile of control state program intensity. Panel a: lowest tertile, Panel b: middle tertile, Panel c: highest tertile.

Panel a

Panel b

Figure S6. RMSE for regression errors for prevalence (Eq. 1) and consumption (Eq. 2), with different discount rates for control expenditures for California and control populations.

The three lines indicate RMSE for various annual discount rates for California; thick line: 0, thin line: 0.05, dashed line: 0.1

## Discussion: Calculations for other estimates from literature

The coefficient for the effect of an additional dollar of cumulative control expenditure per year on per capita consumption in [23] is expressed, in terms of the ECTCP model [1] as

$\frac{\partial c}{\partial E}= \alpha_{1}^{'}*\hat{{cps}_{CA}}+ \beta_{1}*\hat{{prev}_{CA}}.$ Eq. S5

The definitions and values used in the calculation of $\frac{\partial c}{\partial E}$ are shown in Table S5.

Table S5. Calculations to convert ECTCP model to predict per capita consumption.

| Variable | Mean | SE | Interpretation |
| --- | --- | --- | --- |
| *α*_1_’ | 0.000503 | 0.000097 | Coefficient for tobacco control expenditure, Eq 1, predicting prevalence, divided by 100 to change measurement scale of prevalence from percentage to proportion. |
| $\hat{{cps}_{CA}}$ | 0.194 | 0.025406 | Predicted prevalence, expressed as a proportion, 1984 to 2000 |
| *β*_1_ | 2.22 | 0.267 | Coefficient for tobacco control expenditure, Eq 2, predicting consumption |
| $\hat{{cps}_{CA}}$ | 446 | 2.54 | Predicted consumption, expressed as a proportion, 1984 to 2000 |

A Monte Carlo simulation using Yasai[15] with 10,000 trials was used to calculate the standard error of

$\frac{\partial c}{\partial E}$ = 0.656, SE$\left( \frac{\partial c}{\partial E} \right)$ = 0.0896

The coefficient estimate for the effect of control expenditure on per capita consumption from the sixth column of Table 3 in Farrelly et al. [23] is 0.29, SE = 0.06, expressed as reduction in prevalence for every dollar of expenditure. Using the study sample midpoint year, 1990, as the base year for control expenditures, the all-item CPI inflator for conversion to 2010 dollars is 1.64. In 2010 dollars the coefficient estimate is 0.476, SE = 0.0984.

The coefficient for the effect of tobacco control expenditure on per capita consumption from Table 4 of Tauras [24] is 0.68, SE = (0.68 / t-score) = (0.68 /2.48 ) = 0.274. The all-item CPI inflator for conversion from 2007 to 2010 dollars is 1.05. In 2010 dollars, the coefficient is 0.714, SE = 0.288.

# Data Appendix

1. **Download Data Files (Key for variable names x_s_n: x is variable name, s is state index, n is time index)**

| **Variable to Download** | | | | |
| --- | --- | --- | --- | --- |
| **Feature Name** | **Description** | **Source or Download Link** | **Source or Download Details** | **Additional Source or Download Details** |
| NIPA nhce | total nominal NIPA health care expenditure by state | <https://www.bea.gov/regional/downloadzip.cfm> | <https://www.bea.gov/regional/index.htm> | Bureau of Economic Analysis NIPA Data on health expenditures, and personal income NIPA Health Care expenditure, personal income, and resident population, annual estimates by individual state and DC Frequency: annual, calendar year  Break in data with years before 1997 using the Standard Industrial Classification (SIC) and after using North American Industrial Classification System (NACIS). These two series will be spliced as explained below.  There are codes to identify the specifics health care expenditure which will need to be broken for some of the calculation for the final file. |
| ec_s_n | nominal Annual tobacco control funding, total | <https://www.cdc.gov/statesystem/index.html> | <https://www.cdc.gov/tobacco/about/osh/index.htm> | Custom Reports>Funding>Best Practices and Funding>Expenditures |
| c_s_n | cigarette consumption per smoker | https://www.cdc.gov/statesystem/index.html | [https://www.cdc.gov/tobacco/about/osh/index.htm](https://www.cdc.gov/statesystem/index.html) | Download file using the following path: Custom Reports>Policy>The Tax Burden on Tobacco>Cigarette Sales OW |
| p_s_n | nominal cigarette price | https://www.cdc.gov/statesystem/index.html | https://www.cdc.gov/tobacco/about/osh/index.htm | Custom Reports>Policy>The Tax Burden on Tobacco>Cigarette Sales OW |
| s_s | state current smoking prevalence | [https://www.cdc.gov/statesystem/index.html](https://www.cdc.gov/tobacco/about/osh/index.htm) | [https://www.cdc.gov/tobacco/about/osh/index.htm](https://www.cdc.gov/statesystem/index.html) | Custom Reports>Tobacco Use – Survey Data>Cigarette Use (Adults)>Current Smoking - BRFSS |
| y_s_n | nominal per capita state personal income | <https://www.bea.gov/regional/downloadzip.cfm> | <https://www.bea.gov/regional/index.htm> | Download the Annual Personal Income By State File |
| ya_s  of_s  om_s  w_s  a_s | Proportion of state population  Young adults and adults, age 18-44  Women, age 45-64 yrs  Men, age 45-64 yrs  Women of childbearing age  Elderly (age >= 64 yrs) | 1980-1990  <https://www2.census.gov/programs-surveys/popest/datasets/1980-1990/state/asrh/st_int_asrh.txt>  1990-1999  <https://www2.census.gov/programs-surveys/popest/tables/1990-2000/intercensal/st-co/stch-icen1990.txt> to  <https://www2.census.gov/programs-surveys/popest/tables/1990-2000/intercensal/st-co/stch-icen1999.txt>  2000-2010  <https://www2.census.gov/programs-surveys/popest/datasets/2000-2010/intercensal/state/st-est00int-agesex.csv>  2010-2019  <https://www2.census.gov/programs-surveys/popest/tables/2010-2018/state/asrh/sc-est2018-alldata5.csv> | 1980-1990  <https://www.census.gov/content/census/en/data/datasets/time-series/demo/popest/1980s-state.html>  1990-1999  <https://www.census.gov/content/census/en/data/datasets/time-series/demo/popest/intercensal-1990-2000-state-and-county-characteristics.html>  2000-2010  <https://www.census.gov/content/census/en/data/datasets/time-series/demo/popest/intercensal-2000-2010-state.html>  2010-2019  <https://www.census.gov/data/datasets/time-series/demo/popest/2010s-state-detail.html> | Download State level inter- and post-censal annual state population estimates by age and sex |
| b_s  hs_s | Proportion of state population  Non-Hispanic Black  Hispanic ethnicity | Annual BRFSS data files 1985-2018 | <https://www.cdc.gov/brfss/annual_data/annual_data.htm> |  |
| mcd_s | Proportion of the state population enrolled in Medicaid | <https://www.cms.gov/Research-Statistics-Data-and-Systems/Statistics-Trends-and-Reports/NationalHealthExpendData/NationalHealthAccountsStateHealthAccountsResidence.html> | Health expenditures by state of residence, 1991-2014 [ZIP, 347KB] | Within zip file, file name MEDICAID_ENROLLMENT14.CSV |
| tbeds_s | Total state hospital beds per 1,000 population | Kaiser Family Foundation Data  <https://www.kff.org/other/state-indicator/beds-by-ownership/?currentTimeframe=0&sortModel=%7B%22colId%22:%22Location%22,%22sort%22:%22asc%22%7D> | 1980 to 1998: annual AHA Hospital Statistics, American Hospital Association  199 to 2017: Kaiser Family Foundation, State Health Facts |  |
| unr_s | State unemployment rate | https://download.bls.gov/pub/time.series/la/ | la.data.2.AllStatesU | Series LAUSTXX0000000000009. Where XX is state FIPS code |
| hsg_s  ctd_s | State control attainment at age 25  High school graduate  College or technical degree | Annual BRFSS data files 1985-2018 | <https://www.cdc.gov/brfss/annual_data/annual_data.htm> |  |
| pov_s | State poverty rate | US Census Bureau | http://www2.census.gov/programs-surveys/cps/tables/time-series/historical-poverty-people/ | File hstpov21.xls |
| aicpu deflators | All item CPI-U index | <https://download.bls.gov/pub/time.series/cu/cu.data.1.AllItems> | Price index files, Consumer Price Index for All Urban Consumers (CPI-U) Dimensions, national and Census Region cross-section, Annual averages, Base year = 100, 1982-1984 3.1.a. all-items, time span 1967-2018 |  |
| mccpu deflator | Medical care CPI-U index | <https://download.bls.gov/pub/time.series/cu/cu.data.15.USMedical> | 3.1.b.i. national average: CUUR0000SA0L5 3.1.b ii. Northeast: CUUR0100SA0L5 3.1.b.iii. Midwest: CUUR0200SA0L5 3.1.b.iv. South: CUUR0300SA0L5 3.1.b.v. West: CUUR0400SA0L5 3.1.c. medical care, time span 1935-2018 national, 1978-2018, Census Region |  |
| ailmcpu deflators | All item less Medicare CPI-U index | <https://download.bls.gov/pub/time.series/cu/cu.data.1.AllItems> | 3.1.a.i. national average: CUUR0000SA0 3.1.a ii. Northeast: CUUR0100SA0 3.1.a.iii. Midwest: CUUR0200SA0 3.1.a.iv. South: CUUR0300SA0 3.1.a.v. West: CUUR0400SA0 3.1.b. all-items less medical care, time span 1979-2018 URL for download for all-items less medical care: |  |
| ailmcspu deflators | Medicare Care Series CPI-U index | <https://download.bls.gov/pub/time.series/cu/cu.data.1.AllItems> | 3.1.c.i. national average: CUUR0000SAM 3.1.c ii. Northeast: CUUR0100SAM 3.1.c.iii. Midwest: CUUR0200SAM 3.1.c.iv. South: CUUR0300SAM 3.1.c.v. West: CUUR0400SAM 3.1.d. Medical care services time span 1984-2018 |  |
| CAaicspu deflators | California all-item CPI-U price index | <http://www.dof.ca.gov/Forecasting/Economics/Eco_Forecasts_Us_Ca/> | Adjustment factors for California price index due to discontinuation of previous BLS sub-state price indices |  |
| CMS nhcep (hp_s_n) | CMS total agg pesonal healthcare expenditure by provider | <https://www.cms.gov/Research-Statistics-Data-and-Systems/Statistics-Trends-and-Reports/NationalHealthExpendData/NationalHealthAccountsStateHealthAccountsResidence.html> | Health expenditures by state of provider, 1980-2014 [ZIP, 278KB] | Within zip file, file name PROV_US_AGGREGATE14.CSV |
| BEA Population | BEA Population | <https://www.bea.gov/regional/downloadzip.cfm> | <https://www.bea.gov/regional/index.htm> |  |
| ec_s_n | State tobacco control expenditures | Unpublished CDC data (1989 to 2007)  California State Tobacco Control program (2012, 2013)  CDC state system (2008-2010, 2014-2016) | <https://www.cdc.gov/statesystem/index.html>  Custom Reports > Funding | Data for 1989 to 2007, and 2012 to 2013 provided by California Dept. of Public Health, Tobacco Control Branch |

1. **Create Deflators (2010 $)**

| **Feature Name** | **Description** | **Calculation** |
| --- | --- | --- |
| mccpu deflator | Medical care CPI-U index | Divide 2010 CPI_U Index by the respective year for each year to calculator ratio which is the deflator |
| aicpu deflators | All item CPI-U index | Divide 2010 CPI_U Index by the respective year for each year to calculator ratio which is the deflator |
| ailmcpu deflators | All item less medicare CPI-U index | Divide 2010 CPI_U Index by the respective year for each year to calculator ratio which is the deflator |
| ailmcspu deflators | Medicare Care Serices CPI-U index | Divide 2010 CPI_U Index by the respective year for each year to calculator ratio which is the deflator |

1. **Transform Data for Final File**

| **Calculated Variables** | | |
| --- | --- | --- |
| **Year** | **Year** | **Formula** |
| hr_s | Total real CMS health care expenditure by state | personal_med_exp * medicare care deflator (2010 $) * (10^-3) |
| y_s | Real per capita state personal income | y_s = y_s_n * all items less medicare care deflator (2010 $) * (10^-4) |
| cps_s | Consumption per smoker by state | cps_s = c_s / s_s |
| cpsa_s | Consumption per adult smoker by state | cps_s = c_s / ( s_s * % of 18 years olds ) % of Population 18 years File |
| p_s | Real cigarette price | p_s = p_s_n * all item 2010 deflator (2010 $) |
| ec_s | Real annual tobacco control funding, total | ec_s = ec_s_n * all item 2010 deflator (2010 $) |
| hp_s | Real CMS total agg personal healthcare expenditure by provider | hp_s = ( (hp_s_n * 1000000) / bea population ) * medicare care deflator (2010 $) * (10^-3) |
| n11_s | Convert NIPA state total per capita health care and social assistance production, both SIC and NAIC data, from nominal dollars to real per capita health care production base year dollars | n11_s = (10^(6 - 3)) * ( ( hlth_care_n_social_assist_70 - social_assist_73 )/sorted_f$bea_pop ) * medical care services deflators (2010 $) |
| n10_s | Convert NIPA state total per capita health care and social assistance production, both SIC and NAIC data, from nominal dollars to real per capita health care production base year dollars | n10_s = (csic_health_srvc_65/ bea population) * medical care services deflators (2010 $) * (10^(6-3)) |
| cec_s | Convert annual real tobacco control expenditure to cumulative expenditure | cec_s(t) = cec_s(t-1) + d*ec_s(t),  t is year, d is discount rate (d>= 0 and <= 1) |
| **k1** | Instrumental Variable 1 | Instrumental Variable |
| **k2** | Instrumental Variable 2 | Instrumental Variable |
| **k3** | Instrumental Variable 3 | Instrumental Variable |
| **k4** | Instrumental Variable 4 | Instrumental Variable |
| **k5** | Instrumental Variable 5 | Instrumental Variable |
| **k6** | Instrumental Variable 6 | Instrumental Variable |
| **k7** | Instrumental Variable 7 | Instrumental Variable |
| **k8** | Instrumental Variable 8 | Instrumental Variable |
| **k9** | Instrumental Variable 9 | Instrumental Variable |
| **k10** | Instrumental Variable 10 | Instrumental Variable |
| **k11** | Instrumental Variable 11 | Instrumental Variable |
| **k12** | Instrumental Variable 12 | Instrumental Variable |
| **k13** | Instrumental Variable 13 | Instrumental Variable |
| **k14** | Instrumental Variable 14 | Instrumental Variable |
| **k15** | Instrumental Variable 15 | Instrumental Variable |
| **k16** | Instrumental Variable 16 | Instrumental Variable |
| **k17** | Instrumental Variable 17 | Instrumental Variable |
| **k18** | Instrumental Variable 18 | Instrumental Variable |
| **k19** | Instrumental Variable 19 | Instrumental Variable |
| **k20** | Instrumental Variable 20 | Instrumental Variable |

# REFERENCES

1. Lightwood, J. and S. Glantz, *The Effect of the California Tobacco Control Program on Smoking Prevalence, Cigarette Consumption, and Healthcare Costs: 1985-2008.* PLoS ONE, 2013. **8**(2): p. e47145.

2. *Chronology of changes in the Consumer Price Index* 2020; Available from: https://www.bls.gov/cpi/additional-resources/historical-changes.htm.

3. *Economic Forecasts, U.S. and California: Consumer Price Index Forecast - Annual & Monthly*. 2020; Available from: https://www.dof.ca.gov/Forecasting/Economics/Eco_Forecasts_Us_Ca/.

4. Phillips, P.C.B. and B.E. Hansen, *Statistical inference in instrumental variables regression with I(1) processes.* The Review of Economic Studies, 1990. **57**(1): p. 99-125.

5. Phillips, P.C.B., *Optimal estimation of cointegrated systems with irrelevant instruments.* Journal of Econometrics, 2014. **178 part 2**: p. 210-224.

6. Montiel Olea, J.L. and C. Flueger, *A Robust test for weak instruments.* Journal of Business & Economic Statistics, 2013. **31**(3): p. 368-369.

7. Pflueger, C. and S. Wang, *A robust test for weak instruments in Stata.* The Stata Journal, 2015. **15**(1): p. 216-225.

8. Lightwood, J.M., A. Dinno, and S.A. Glantz, *Effect of the California tobacco control program on personal health care expenditures.* PLoS Medicine, 2008. **5**(8): p. e178.

9. Maddala, G.S. and I.-M. Kim, *Unit Roots, Cointegration, and Structural Change*. 1998, Cambridge: Cambridge University Press.

10. Kwiatkowski, D., et al., *Testing the null hypothesis of stationarity against the alternative of a unit root: how sure are we that economic times series have a unit root?* Journal of Econometrics, 1992. **54**(1-3): p. 159-178.

11. Hobijn, B., P.H. Franses, and M. Ooms, *Generalizations of the KPSS-test for Stationarity*. 1998, Econometric Institute, Erasmus University: Rotterdam, Netherlands.

12. The SAS Institute Inc., *SAS*. 2016: Cary, NC.

13. The R Foundation for Statistical Computing, *R, version 3.4.2, <https://www.r-project.org/foundation>*. 2017.

14. StataCorp LP, *Stata version 16*. 2019, College Station, Texas.

15. Eckstein, J. and S. Riedmuller, *Yasai*. 2019, Rutgers Business School, Rutgers University: New Brunswick, NJ.

16. Abadie, A., A. Diamond, and J. Hainmueller, *Synthetic control methods for comparative case studies: Estimating the effect of California’s Tobacco Control Program.* Journal, American Statistical Association, 2010. **105**(490): p. 493-505.

17. Hatanaka, M., *Time-Series-Based Econometrics*. 1996, New York NY, Oxford UK: Oxford University Press.

18. StataCorp LP, *Arfima -Autoregressive fractionally integrated moving-average models*, in *STATA Time Series Reference Manual, Release 16*. 2019, Stata Press: College Station, Texas. p. 319-329.

19. Elliott, G., *On the robustness of cointegration methods when regressors almost have unit roots.* Econometrica, 1998. **66**(1): p. 149-158.

20. Centers for Disease Control and Prevention (CDC). *State Tobacco Activities Tracking and Evaluation (STATE) System, Tobacco Use - Survey Data*. 2021 [cited 2021 Dec 21]; Available from: https://nccd.cdc.gov/STATESystem/rdPage.aspx?rdReport=OSH_State.CustomReports.

21. Dai, H. and A.M. Leventhal, *Prevalence of e-Cigarette Use Among Adults in the United States, 2014-2018.* JAMA, 2019. **322**(18): p. 1824-1827.

22. Sapru, S., et al., *E-cigarettes use in the United States: reasons for use, perceptions, and effects on health.* Bmc Public Health, 2020. **20**(1).

23. Farrelly, M.C., T.F. Pechacek, and F.J. Chaloupka, *The impact of tobacco control program expenditures on aggregate cigarette sales: 1981-1998.* Journal of Health Economics, 2001. **22**(203): p. 843-859.

24. Tauras, J.A., et al., *State tobacco control expenditures and tax paid cigarette sales.* PLoS One, 2018. **13**(4): p. e0194914.
