# Supplementary material for: Smoking and healthcare expenditure reductions associated with the California Tobacco Control Program, 1989 to 2019: A predictive validation
Source: PLoS One. 2023 Mar 16;18(3):e0263579. doi: 10.1371/journal.pone.0263579 (PMC10019627; doi:10.1371/journal.pone.0263579)
Supplement: S1 File — (DOCX) [file pone.0263579.s003.docx]

/* commands for main regression results*/

/* declare time series format */

tsset year

/* differences between California and control state population */

gen ds = s_s_c - s_s

gen dcpsa = cpsa_s_c - cpsa_s

gen dcps = cps_s_c - cps_s

gen de = cec_sa - cec_sa_c

gen dprc = p_sa - p_sa_c

gen dy = y_sa - y_sa_c

/* measurement model for 2011 break in BRFSS survey design */

gen d2011 = 0

replace d2011 = 1 if year >= 2011

gen s_s_c11 = s_s_c*d2011

gen cpsa_s_c11 = cpsa_s_c*d2011

gen ds11 = ds*d2011

gen dcpsa11 = dcpsa*d2011

/* re-estimation of published model using updated data to 2008 */

constraint 1 s_s_c = 1

reg3 (s_s = s_s_c L.de L.dprc L.dy) if year >= 1985 & year <= 2008, constraints(1) inst(k1-k20) 2sls

reg3 (ds = L.de L.dprc L.dy) if year >= 1985 & year <= 2008, inst(k1-k20) 2sls

constraint 2 cpsa_s_c = 1

reg3 (cpsa_s = cpsa_s_c L.de L.dprc L.dy) if year >= 1985 & year <= 2008, constraints(2) inst(k1-k20) 2sls

reg3 (dcpsa = L.de L.dprc L.dy) if year >= 1985 & year <= 2008, constraints(2) inst(k1-k20) 2sls

ivregress 2sls n1011_s (n1011_s_c L.ds L.dcpsa L.dy = k1-k20) if year >= 1985 & year <= 2008

ivregress 2sls hp_s (hp_s_c L.ds L.dcpsa L.dy = k1-k20) if year >= 1985 & year <= 2008

/* published model estimated with updated data */

constraint 1 s_s_c = 1

reg3 (s_s = s_s_c d2011 s_s_c11 L.de L.dprc L.dy) if year >= 1985 & year <= 2018, constraints(1) inst(k1-k20) 2sls

gen dsiv = s_s_c - s_s

reg3 (dsiv = d2011 s_s_c11 L.de L.dprc L.dy) if year >= 1985 & year <= 2018, inst(k1-k20) 2sls

constraint 2 cpsa_s_c = 1

reg3 (cpsa_s = cpsa_s_c d2011 cpsa_s_c11 L.de L.dprc L.dy) if year >= 1985 & year <= 2018, constraints(2) inst(k1-k20) 2sls

gen dcpsaiv = cpsa_s_c - cpsa_s

reg3 (dcpsaiv = d2011 cpsa_s_c11 L.de L.dprc L.dy) if year >= 1985 & year <= 2018, inst(k1-k20) 2sls

ivregress 2sls n1011_s (n1011_s_c L.ds L.dcpsa L.ds11 L.dcpsa11 L.dy = k1-k20) if year >= 1985 & year <= 2017

ivregress 2sls hp_s (hp_s_c L.ds L.dcpsa L.ds11 L.dcpsa11 L.dy = k1-k20) if year >= 1985 & year <= 2014

/* forecast model estimated with updated data */

constraint 1 L.s_s_c = 1

reg3 (s_s = L.s_s_c d2011 L.s_s_c11 L.de L.dprc L.dy) if year >= 1985 & year <= 2018, constraints(1) inst(k1-k20) 2sls

gen dsLiv = L.s_s_c - s_s

reg3 (dsLiv = d2011 L.s_s_c11 L.de L.dprc L.dy) if year >= 1985 & year <= 2018, inst(k1-k20) 2sls

constraint 2 L.cpsa_s_c = 1

reg3 (cpsa_s = L.cpsa_s_c d2011 L.cpsa_s_c11 L.de L.dprc L.dy) if year >= 1985 & year <= 2018, constraints(2) inst(k1-k20) 2sls

gen dcpsaLiv = L.cpsa_s_c - cpsa_s

reg3 (dcpsaLiv = d2011 L.cpsa_s_c11 L.de L.dprc L.dy) if year >= 1985 & year <= 2018, inst(k1-k20) 2sls

ivregress 2sls n1011_s (L.n1011_s_c L.ds L.dcpsa L.ds11 L.dcpsa11 L.dy = k1-k20) if year >= 1985 & year <= 2017

ivregress 2sls hp_s (L.hp_s_c L.ds L.dcpsa L.ds11 L.dcpsa11 L.dy = k1-k20) if year >= 1985 & year <= 2014
